# Associations of maternal angiogenic factors during pregnancy with childhood carotid intima-media thickness and blood pressure

**DOI:** 10.1016/j.atherosclerosis.2021.11.005

**Published:** 2021-11-06

**Authors:** Meddy N. Bongers-Karmaoui, Vincent W.V. Jaddoe, Romy Gaillard

**Affiliations:** a The Generation R Study Group, Erasmus University Medical Center, Rotterdam, the Netherlands; b Department of Pediatrics, Sophia Children’s Hospital, Erasmus University Medical Center, Rotterdam, the Netherlands

**Keywords:** Maternal PlGF, Maternal sFlt-1, Pregnancy, Childhood vascular development

## Abstract

**Background and aims:**

Reduced maternal placental growth factor (PlGF) and higher soluble fms-like tyrosine kinase (sFlt-1) concentrations in pregnancy may have persistent effects on offspring vasculature. We hypothesized that suboptimal maternal angiogenic factors in pregnancy may adversely affect fetal vascular development, leading to an increased risk of adverse atheriosclerotic adaptations and higher blood pressure in offspring.

**Methods:**

In a population-based prospective cohort among 4565 women and their offspring, we examined the associations of maternal serum PlGF and sFlt-1 concentrations in the first half of pregnancy with offspring vascular development. We measured childhood blood pressure and obtained childhood carotid intima media thickness and carotid distensibility through ultrasonography at 9 years.

**Results:**

After adjustment for maternal sociodemographic and lifestyle characteristics, no associations were present of maternal first and second trimester angiogenic factors with childhood blood pressure, carotid intima media thickness (IMT) or distensibility in the total population. In preterm born children only, higher maternal second trimester PlGF concentrations, but not sFlt-1 concentrations, were associated with a lower childhood diastolic blood pressure (difference: -0.16 SDS (95% CI -0.30, –0.03) per SDS increase in maternal second trimester PlGF concentration). No associations among children born small-for-gestational age were present.

**Conclusions:**

In a low-risk population, maternal angiogenic factors in the first half of pregnancy are not associated with childhood blood pressure, carotid IMT or carotid distensibility after considering maternal socio-demographic and lifestyle factors. Only in children born preterm, lower maternal second trimester PlGF concentrations are associated with higher childhood diastolic blood pressure, but not with other vascular outcomes.

## 1 Introduction

Placental vascular development and function are major determinants of fetal cardiovascular development. Abnormalities in placental vascular development and function may lead to long-term alterations in childhood vasculature [1]. A normal fetal vascular development, and subsequent healthy cardiovascular structure and function in later life, requires an adequate feto-placental angiogenesis and vascularization, which is regulated by a balance of pro- and anti-angiogenic factors [2–4].

Placental growth factor (PlGF), a pro-angiogenic factor, and its soluble receptor fms-like tyrosine kinase (sFlt-1), an anti-angiogenic factor, are produced by placental cytotrophoblasts during pregnancy and are among the most important circulating angiogenic factors in pregnancy [5–7]. Impaired maternal angiogenic factor concentrations may lead to impaired remodeling of the spiral arteries and a disrupted vascular development of the feto-placental villous tree, which may lead to alterations in fetal vascular development [8–10]. A poor feto-placental vascularization may lead to a rise in fetal vascular intraluminal pressure, which damages the vascular walls and causes arteriosclerosis. This might increase the intima media thickness and reduce distensibility of smaller and larger vasculature, predisposing to cardiovascular dysfunction later in life [11,12].

Previously, we found that suboptimal feto-placental vascular flow, characterized by a higher third trimester umbilical artery pulsatility index, is associated with a higher childhood systolic blood pressure and lower left ventricular mass. Stronger associations tended to be present among girls as compared with boys. No associations with offspring diastolic blood pressure were present [13]. We have also shown that imbalanced maternal angiogenic factor concentrations in the first half of pregnancy are associated with narrower childhood retinal arteriolar, but not venular, caliber [14,15]. These previous findings suggest that maternal angiogenic factors affect childhood microvasculature. However, not much is known about the influence of maternal angiogenic factors during pregnancy on the development of the larger vessels and blood pressure in the offspring. Among higher-risk populations, including children born small for their gestational age and children born preterm, studies have shown that an impaired feto-placental vascular function is related to changes in large vessel structures. A prospective study among 44 adolescents, found that abnormal feto-placental blood flow among fetuses, who were small for their gestational age (SGA), was associated with smaller end-diastolic vessel diameters of the abdominal aorta and popliteal artery in young adulthood [16]. These associations might be stronger among children born preterm. A study in 64 extreme preterm born children (birth at a mean gestational age of 27 weeks) showed that preterm birth due to placental dysfunction had a stronger association with childhood structural changes in the arterial wall at 7 years, than preterm birth alone [17]. Another study in 72 preterm (born <37 weeks of gestation) born adults showed that preterm born offspring of hypertensive pregnancies have a higher carotid intima-media thickness than children born preterm from normotensive pregnancies [18].

We hypothesized that lower maternal PlGF and higher sFlt-1 concentrations in the first half of pregnancy may adversely affect fetal vascular development, leading to increased risk of adverse atheriosclerotic adaptations, predisposing offspring to a higher blood pressure in later life. These effects may be most pronounced among children exposed to a more adverse intra-uterine environment who are subsequently born preterm or small for their gestational age. Therefore, we examined, in a population based prospective cohort study among 4565 mothers and their children, the associations of maternal first and second trimester PlGF and sFlt-1 concentrations with childhood carotid intima media thickness, carotid distensibility and blood pressure at 9 years. As a secondary step, we specifically explored whether these associations were different among children born preterm, born small for their gestational age or were modified by offspring gender.

## 2 Materials and methods

### 2.1 Study design and subjects

**Fig. 1:**
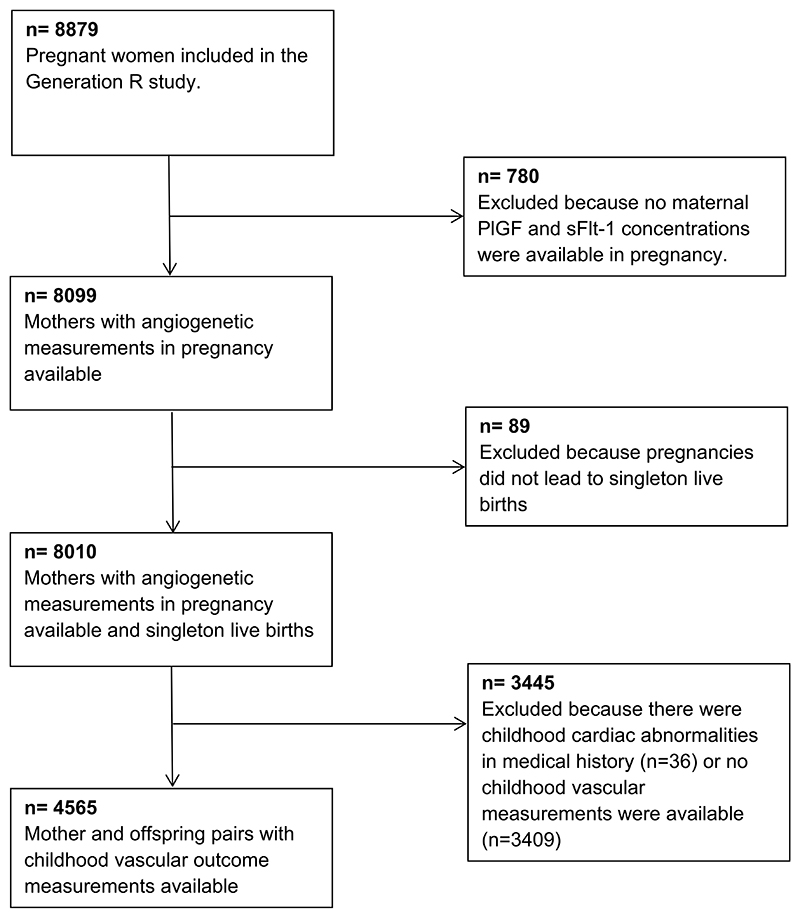
Flow chart of the study participants.

This study was embedded in the Generation R Study, a population-based prospective cohort study from early pregnancy onwards in Rotterdam, the Netherlands [20]. Approval for the study was obtained from the Medical Ethical Committee of Erasmus MC University Medical Center Rotterdam, The Netherlands. Written consent was obtained from all participants. In total, 8879 pregnant women were enrolled between 2001 and 2005. We excluded mothers without PlGF and sFlt-1 concentrations available in early pregnancy (n = 780) and pregnancies not leading to singleton live births (n = 89), leading to 8010 mothers and their children available for analysis. After exclusion of children without available blood pressure measurements or good quality carotid ultrasound measurements, our final population for analyses consisted of 4565 mothers and their children (Fig. 1).

### 2.2 Maternal PlGF and sFlt-1 concentrations

In the first trimester (median 13.2, 95% range 9.6, 7.6 weeks) and second trimester (median 20.4, 95% range 18.5, 23.5 weeks) maternal venous blood plasma samples were drawn [19–21]. Blood samples were centrifuged and thereafter stored at –80 °C. sFlt-1 and PlGF concentrations were analyzed using a prototype of a microparticle-enhanced immunoassay on the Architect System (Abbott Diagnostics B.V., Hoofddorp, the Netherlands). The between-run coefficients of variation for plasma sFlt-1 were 2.8% at 5.5 ng/mL and 2.3% at 34.0 ng/mL; and 4.7% at 24 pg/mL, and 3.8% at 113 pg/mL for plasma PlGF. The highest detected level was 150 ng/mL for sFlt-1 and 1500 pg/mL for PlGF [21]. The sFlt-1/PlGF ratio was calculated by dividing the sFlt-1 concentration by the PlGF concentration. Because these measurements were not normally distributed, we log-transformed them for further analyses. Maternal PlGF and sFlt-1 concentrations and sFlt-1/PlGF ratio were categorized as quintiles and analyzed across the full range after construction of standard deviation (SDS) scores.

### 2.3 Vascular measurements

At the age of 9 years (mean 9.8, SD 0.3), systolic and diastolic blood pressures (mmHg) were measured at the right brachial artery, four times with 1-min intervals, using the validated automatic sphygmanometer Datascope Accutor Plus TM (Paramus, NJ) [24]. Mean systolic and diastolic blood pressure values were calculated, using the last three blood pressure measurements.

To make ultrasonographic recordings of the common carotid artery for carotid IMT and distensibility measurements, we used the Logiq E9 (GE Medical Systems, Wauwatosa, WI, USA). Children were in the supine position with the head tilted in the contralateral direction. The common carotid artery was identified in a longitudinal plane, ~10 mm proximal from the carotid bifurcation. We obtained three recordings on both sides that included the coinciding cardiac cycles. Measurements were performed offline in a semi-automatic manner using Carotid Studio (Cardiovascular Suite (Quipu srl, Pisa Italy)). The recording was frozen on each R-wave of the ECG, the carotid IMT was then measured at the far wall as the average distance between the lumen-intima and the media-adventitia interfaces and the average of all frames was computed. Carotid distensibility was defined as the relative change in lumen area during systole for a given pressure change. The lumen diameter was automatically computed as the average distance between the far and near media-adventia interfaces for each frame of the acquired image sequence. The distension was calculated as the difference between the diastolic and systolic lumen diameter for each cardiac cycle in the recording. The average distension and diameter were used to calculate distensibility. In a reproducibility study performed among 47 subjects, the interobserver and intraobserver intraclass correlation coefficient were greater than 0.85 for distensibility and 0.94 for IMT.

We included all children with at least one successful carotid IMT or distensibility measurement in the analyses. We calculated the overall mean carotid IMT (mm) and distensibility (kPa-1*10-3). For the final analyses, distensibility was log-transformed to deal with a skewed distribution.

### 2.4 Covariates

Information on maternal age, ethnicity, educational level, parity, folic acid supplement use and weight just before pregnancy was obtained at enrolment through questionnaires [22]. Maternal height was measured at intake without shoes. Body mass index (BMI) was calculated. Maternal smoking status during pregnancy was assessed through questionnaires in each trimester [22]. Information on gestational hypertensive disorders and child’s sex, gestational age and weight at birth was obtained from medical records [23–25]. Small for gestational age at birth (SGA) and large for gestational age at birth (LGA) were defined as a sex and gestational age adjusted birth weight below the 10th percentile and above 90th percentile in the study cohort, respectively. Gestational age was divided in preterm (<37 weeks of gestation) and term birth (≥37 weeks of gestation). At the age of 9 years, we calculated child BMI using height and weight measured without shoes and heavy clothing.

### 2.5 Statistical analyses

First, we performed 2 non-response analyses to compare population characteristics of mothers with and without placental angiogenic factors available and mothers and children participating in the follow-up study to those without. We compared population characteristics of specific subgroups of interest. Second, we assessed the associations of maternal first and second trimester PlGF and per SDS change with childhood systolic and diastolic blood pressure, carotid IMT and distensibility using linear regression models. We constructed two models based on previous literature and a Directed Acyclic Graph (DAG) analysis to identify which factors may act as confounders or potential mediators (Supplementary Fig. 1) [1]: basic model, adjusted for gestational age at blood sampling, child’s age and sex [2]; confounder model, which was considered as the main model. The confounder model is the basic model additionally adjusted for maternal socio-demographic and lifestyle factors. Variables were selected based on their associations with exposures and outcomes of interest, or change in effect estimates >10% in our study sample. Using these criteria, we included gestational age at intake, gestational age at blood sampling, maternal age, prepregnancy BMI, educational level, ethnicity, smoking and alcohol consumption during pregnancy, folic acid supplement use and child’s age and sex as possible confounders. Based on biological hypotheses from previous literature [26–28], we examined whether the associations of maternal plasma PlGF and sFlt-1 concentrations with childhood vascular outcomes were modified by fetal sex, gestational age at birth and gestational-age-adjusted birth weight by examining the statistical interaction terms. As significant interactions of maternal angiogenic factors with gestational age at birth and gestational-age-adjusted birthweight were present for childhood vascular outcomes, we performed further subgroup analyses in children born preterm and term and SGA, AGA and LGA. As gestational adjusted birthweight could be a mediator in the association between maternal angiogenic factors and childhood vascular outcomes among preterm born children, we additionally further adjusted any significant associations for gestational age and sex adjusted birthweight. To reduce selection bias due to missing data, we used multiple imputations for missing covariates [29]. The analyses were performed using the Statistical Package of Social Sciences version 24.0 for Windows (SPSS Inc. Chicago, IL, USA).

## 3 Results

### 3.1 Population characteristics

Table 1 shows the population characteristics. Median maternal PlGF concentrations were 41.3 pg/ml (95% range 14.6, 191.2 pg/ml) in the first trimester and 196.0 pg/ml (95% range 73.8, 602.7 pg/ml) in the second trimester. Median maternal sFlt-1 concentrations were 5.7 pg/ml (95% range 1.9, 13.6 pg/ml) in the first trimester and 4.9 pg/ml (95% range 1.5, 16.6 pg/ml) in the second trimester. Non-response analysis showed that women with serum angiogenic factors available were more often Dutch or European and were higher educated, compared to women without angiogenic factors available. Child characteristics from children with mothers with serum angiogenic factors available did not differ from child characteristics from children with mothers without serum angiogenic factors available (Supplementary Table S1). No differences in angiogenic factors of mothers with children who participated in vascular follow-up measurements as compared to those who did not participate in follow-up measurements were present (Supplementary Table S2).

The subgroup of children born small for their gestational age (SGA) consisted of 455 children with a median gestational age at birth of 39.9 (35.3–42.1) weeks, which was lower than in children born AGA (40.1 (35.9–42.4) weeks) and LGA (40.4 (36.3–42.4) weeks) (Supplementary Table S3). Mean birthweight of the SGA born children was 2641 (364) gram *versus* 3432 (428) gram in AGA born children and 4283 (363) gram in LGA born children. Mean systolic and diastolic blood pressure was not different between children born SGA and children born AGA and LGA. Mean childhood carotid IMT was lower and carotid distensibility was higher in children born SGA. In children born LGA, mean carotid distensibility was lower than in children born AGA. No other differences in mean childhood vascular outcomes were present between children born LGA and AGA.

Our subgroup of preterm born children consisted of 206 children with a median gestational age at birth of 35.6 (27.4–36.9) weeks (Supplementary Table S4). In 88.4% of all preterm born children, the delivery started spontaneously. This was not different from the term born children (86.4%). Mean birthweight of the preterm born children was 2318 (615) gram *versus* 3491 (493) gram in term born children. Mean systolic and diastolic blood pressure, carotid IMT and distensibility in childhood were not different between the term and preterm born children.

The total population consisted of 2321 girls and 2244 boys. Girls had lower maternal second trimester PlGF concentrations and higher maternal first and second trimester sFlt-1 concentrations. Girls had a slightly higher systolic blood pressure and diastolic blood pressure, lower carotid IMT and higher distensibility as compared to boys (*p*-value<0.05) (Supplementary Table S5).

### 3.2 Associations of maternal angiogenic factors with childhood vascular outcomes

In the basic model, higher maternal first trimester sFlt-1 concentrations and higher sFlt-1/PlGF ratio were associated with a lower child-hood systolic blood pressure (*p*-value<0.05) (Table 2). Higher maternal first trimester PlGF concentrations and lower sFlt-1/PlGF ratio were associated with a higher childhood diastolic blood pressure (*p*-value<0.05). In second trimester, only lower maternal first trimester sFlt-1 concentrations were associated with a higher childhood systolic blood pressure (*p*-value<0.05). However, all associations were fully explained by adjustment for other maternal sociodemographic and lifestyle characteristics in the confounder model. No associations were found with childhood cIMT or distensibility. No consistent associations of maternal first and second trimester PlGF or sFlt-1 concentrations quintiles and PlGF/sFlt-1 ratio quintiles with childhood systolic blood pressure or diastolic blood pressure, cIMT or distensibility were present (Supplemental Figs. 2–7).

### 3.3 Subgroup analysis

Significant interactions of maternal angiogenic factors with gestational age at birth and gestational age adjusted birth weight were present for childhood vascular outcomes (*p*-values for the interactions are shown in Supplementary Table s5. Subgroup analysis showed that no distinct differences were found in the associations of maternal angiogenic factors with childhood vascular outcomes among children born SGA, AGA or LGA (Table 3). In preterm born children only, a higher maternal second trimester PlGF concentration was associated with a lower childhood diastolic blood pressure (difference: -0.16 SDS (95% CI -0.30, –0.03) per SDS increase in maternal second trimester PlGF concentration). Also a higher maternal second trimester sFlt-1/PlGF ratio was associated with a higher childhood diastolic blood pressure (difference: 0.19 SDS (95% CI 0.06, 0.16) per SDS increase in maternal sFlt-1/PlGF ratio) (Table 4). These associations were not explained by additional adjustment for gestational age and sex adjusted birthweight. No other associations of maternal angiogenic factors with childhood vascular outcomes were present.

## 4 Discussion

In this population-based cohort study among low-risk pregnant women, we observed no associations of maternal first or second trimester angiogenic factors across the full range with childhood blood pressure, carotid IMT and distensibility, after considering maternal socio-demographic and lifestyle factors. Only in children born preterm, an imbalance in maternal second trimester angiogenic factors, characterized by lower PlGF concentrations and higher sFlt-1/PlGF ratio, is associated with a higher childhood diastolic blood pressure, but not with other vascular outcomes.

### 4.1 Interpretation of the main findings

An increasing amount of evidence suggests that a suboptimal fetoplacental vascular development and function may lead to alterations in fetal vascular development, which may predispose to an increased risk of cardiovascular diseases in later life [1,30]. However, the underlying mechanisms remain poorly understood.

Vasculogenesis and angiogenesis, which are regulated by pro- and anti-angiogenic factors, are the most important processes in the formation of the vascular network in the placenta and the fetus. Based on animal studies, a disrupted balance in maternal pro- and anti-angiogenic factors in pregnancy can lead to fetal vascular adaptations via multiple mechanisms. These mechanisms include direct effects of angiogenic factors on the development of the feto-placental villous tree and fetal blood pressure, and secondary adverse effects on fetal vascular development due to altered feto-placental blood flow and hypoxia [31–33]. A study in Denmark among 2217 mother-offspring pairs showed that higher maternal third trimester sFlt-1 concentrations, but not PlGF concentrations, were associated with lower offspring systolic blood pressure from 4 months to 5 years [34]. This association was found even after adjustment for maternal socio-demographic and lifestyle factors and offspring birth characteristics.

We hypothesized that also an imbalance in maternal angiogenic factors, reflected by lower maternal PlGF concentrations and higher trimester sFlt-1 concentrations, already in the first half of pregnancy, may increase childhood carotid IMT and decrease the arterial distensibility, leading to a higher blood pressure in childhood and adulthood. Predominantly in the first half of pregnancy, trophoblast migration into the spiral arteries causes a dilation of maternal spiral arteries and development of villous tree leading to a favorable uteroplacental vascular resistance [32,33]. Also, first trimester includes the embryonic and early fetal phase, which is essential for development of the fetal cardiovascular system [34]. In this study, we observed no consistent associations of maternal angiogenic factors in the first half of pregnancy with childhood vascular outcomes, after adjustment for other maternal socio-demographic and lifestyle related characteristics. Differences between our study and the Danish study may be related to the timing of maternal angiogenic measurements and offspring vascular assessment. In the second half of pregnancy, the balance in angiogenic factors changes as maternal PlGF concentrations decrease and sFlt-1 concentrations and sFlt-1/PlGF ratio further increase, and may have different effects on offspring vascular development [35]. Also, alterations in offspring vasculature that originate prenatally may still be present in early childhood, but may diminish at a later age due to cardiovascular adaptations. Further studies are needed to examine the associations of maternal angiogenic factors throughout pregnancy with offspring vascular development from early-childhood until adulthood.

Several previous studies have mainly reported effects of intra-uterine fetal growth restriction (IUGR) due to impaired placental function on fetal vascular outcomes [11,16,35]. It is well established that IUGR is characterized by an increased resistance to blood flow in the umbilical artery resulting in an increased workload for the developing fetal cardiovascular system [36]. In Sweden, a prospective study among 21 adolescents with severe IUGR and 23 adolescents with normal fetal growth and normal fetal aortic blood flow examined the association of IUGR with vascular outcomes at the age of 18. All IUGR patients had absent or reverse fetal aortic diastolic blood flow due to an increased placental impedance and/or a birthweight of >2.5 SD below the mean weight of the normal population. This study found that IUGR and abnormal fetal blood flow were associated with smaller end-diastolic vessel diameters of the abdominal aorta and popliteal artery in young adulthood. These findings were not explained by body surface area or sex. No differences were found in arterial stiffness [16]. Another study in 77 pregnant women in Italy found that aortic IMT was significantly higher in IUGR (weight < p10 and umbilical artery pulsatility index >2SD) fetuses and infants compared to healthy controls, both in utero and at a mean postnatal age of 18 months. Systolic blood pressure was higher in offspring with severe IUGR [37]. Fetal aortic IMT was assessed by a Spanish study in 49 AGA, 40 SGA and 35 IUGR fetuses at a mean gestational age of 34 weeks. IUGR was diagnosed when fetal weight was <p10 and umbilical artery pulsatility index >95th percentile. They found a higher aortic IMT in IUGR fetuses compared to SGA and AGA fetuses. No differences in aortic IMT between AGA and SGA fetuses were found, suggesting that the increase in aortic IMT is not present in all small fetuses, but only in those with an abnormal feto-placental blood flow [38]. In our study, we did not find an association of maternal angiogenic factors with childhood vascular outcomes in children born SGA. However, in contrast to these previous studies, our study population included children with a less severe fetal growth restriction defined at birth. Reversed flow in the umbilical artery in pregnancy was not present in our group of SGA born children. Possibly the associations of suboptimal maternal angiogenesis factors with offspring vascular development are only present in children who suffered from a more extreme intra-uterine growth retardation in combination with impaired placental function in the second half of pregnancy.

We observed that, in children born preterm only, higher maternal PlGF concentrations were associated with a lower childhood diastolic pressure. No associations were present with cIMT or distensibility. Even though this finding may reflect a chance finding, within our study cohort we previously showed that higher maternal second trimester PlGF concentrations, but not sFlt-1, were associated with wider offspring retinal arteriolar caliber at 6 years of age. Possibly, these changes in maternal second trimester angiogenic factors reflect a more favorable branching of offspring microvasculature, resulting in a lower childhood diastolic blood pressure, whereas no effects on larger vessels are present among preterm born children [14]. Previous studies have shown that children born preterm have a distinct vascular development from children born at term. Prematurity is associated with changes in the microvasculature, endothelial dysfunction and higher blood pressure in childhood and adolescence [39–41]. Based on these studies, preterm born children might be more prone to differences in vascular development in response to an imbalance in maternal angiogenic factors during pregnancy. Future studies are needed to further investigate these associations for extreme prematurity and less severe prematurity and to explore potential underlying mechanisms using detailed measurements of large and small vascular development and function.

Our findings do not suggest consistent associations of maternal first and second trimester PlGF and sFlt-1 concentrations with childhood arterial vascular development or blood pressure within a low-risk population. However, our study population is a relatively healthy population and is not at risk for severe childhood vascular alterations. Further research in higher-risk populations, including offspring born from mothers with gestational hypertensive disorders, born preterm or with intra-uterine fetal growth restriction is needed to examine whether maternal angiogenic factors are related to offspring vascular health. These studies should focus on offspring arterial vasculature and microvasculature development from early-childhood into adulthood and use advance imaging techniques to enable detailed measurements of sub-clinical vascular changes.

### 4.2 Strengths and limitations

The main strengths of this study were the prospective design with data collection already from early-pregnancy onwards and the large sample size. The response rate at baseline for participation in the Generation R Study cohort was 61%. From the mothers with singleton-life births and available information on the exposures during pregnancy, 57% of the children participated in the current study. A selective non-response could have led to biased effect estimates if associations would be different between the included children and non-included children, this does not seem likely. However, the selection towards a relatively healthy, high-educated population may affect the generalizability of our findings. Approximately, 8% of the children did not have all three carotid IMT measurements at both sides and 19% did not have all three distensibility measurements on both sides. This was due to low quality recordings or missing coinciding cardiac cycles. We included all children with at least one reliable carotid IMT or distensibility measurement in our main analyses. When we repeated the analyses among children with all three measurement on both sides available, we observed similar results (results not shown). As our outcomes are strongly related to each other, we did not correct for multiple testing. However, as we did perform multiple analyses, our findings which show that suboptimal maternal angiogenic factor concentrations in the second trimester are associated with a higher blood pressure in children born preterm only may reflect a chance finding. Further studies are needed to replicate our findings among preterm born children. Finally, although we adjusted for multiple confounding factors, there might still be residual confounding as in any observational study.

### 4.3 Conclusion

Among low-risk pregnant women, no associations of maternal first or second trimester angiogenic factors across the full range with childhood blood pressure, carotid IMT and distensibility were present, after considering maternal socio-demographic and lifestyle factors. Only in children born preterm, an imbalance in maternal second trimester angiogenic factors, characterized by lower PlGF concentrations and higher sFlt-1/PlGF ratio, was associated with a higher childhood diastolic blood pressure, but not with other vascular outcomes. Further studies are needed to examine whether in higher-risk populations maternal angiogenic factors are related to offspring vascular development.

## Supplementary Material

Supplementary Material

## Figures and Tables

**Table 1:** Characteristics of mothers and their children in the Generation R study (n = 4565).

Maternal characteristics	
Age at enrolment, mean (SD), years	30.7 (4.9)
Gestational age at intake, median (95%), weeks	14.1 (10.4–22.5)
Prepregnancy BMI, median (95%), kg/m^2^	22.6 (18.1–34.6)
Parity, no. nulliparous (%)	2665 (58.7)
Ethnicity, no. Dutch or European (%)	2918 (65.1)
Education level, n high (%)	2174 (50.1)
Smoking during pregnancy, n yes (%)	632 (15.4)
Folic acid supplement use, n yes (%)	1625 (46.2)
Pregnancy induced hypertension, n yes (%)	177 (4.0)
First trimester PlGF, median (95%), pg/ml	41.3 (14.6–191.2)
First quintile, pg/ml	7.6–26.1
Second quintile, pg/ml	26.1–35.4
Third quintile, pg/ml	35.4–48.8
Fourth quintile, pg/ml	48.8–79.1
Fifth quintile, pg/ml	79.1–638.2
Second trimester PlGF, median (95%), pg/ml	196.0 (73.8–602.7)
First quintile, pg/ml	10.1–131.3
Second quintile, pg/ml	131.3–171.6
Third quintile, pg/ml	171.6–222.9
Fourth quintile, pg/ml	222.9–308.5
Fifth quintile, pg/ml	308.5–1408.7
First trimester sFlt-1, median (95%), pg/ml	5.7 (1.9–13.6)
First quintile, pg/ml	0.1–3.3
Second quintile, pg/ml	3.3–4.4
Third quintile, pg/ml	4.4–5.7
Fourth quintile, pg/ml	5.7–7.6
Fifth quintile, pg/ml	7.6–30.7
Second trimester sFlt-1, median (95%), pg/ml	4.9 (1.5–16.6)
First quintile, pg/ml	0.0–2.9
Second quintile, pg/ml	2.9–4.2
Third quintile, pg/ml	4.2–5.8
Fourth quintile, pg/ml	5.8–8.2
Fifth quintile, pg/ml	8.2–62.7
**Child characteristics**	
Age, mean (SD), years	9.8 (0.3)
Gender, n female (%)	2321 (50.8)
Birth weight, mean (SD), grams	3438.4 (2250.0–4484.9)
Gestational age at birth, median (95%), weeks	40.1 (35.9–42.3)
BMI, median (95%), kg/m^2^	17.0 (14.0–24.9)
Systolic blood pressure, mean (SD), mmHg	103.2 (7.9)
Diastolic blood pressure, mean (SD), mmHg	58.6 (6,4)
Carotid intima-media thickness, median (95%), mm	0.46 (0.37–0.54)
Carotid distensibility, median (95%), 10-3*kPa-1	55.8 (37.1–85.4)

Values are observed data and represent means (SD), medians (95% range) or numbers of subjects (valid %).

**Table 2:** Associations of maternal first and second PlGF and sFlt-1 concentrations with childhood vascular outcomes at 9 years.

Maternal angiogenesis factors	Difference in childhood systolic blood pressure SDS (95%CI)	*p*-value	Difference in childhood diastolic blood pressure SDS (95%CI)	*p*-value	Difference in childhood intima-media thickness (cIMT) SDS (95%CI)	*p*-value	Difference in childhood distensibility SDS (95% CI)	*p*-value
**First trimester**								
**Maternal PlGF concentrations**								
Basic model^b^	0.01 (−0.04, 0.06)	0.67	0.05 (0.01, 0.10) ^a^	0.03^a^	−0.01 (−0.06-0.05)	0.86	0.01 (−0.04, 0.06)	0.78
Confounder model^c^	−0.01 (−0.06, 0.04)	0.63	0.03 (−0.02, 0.08)	0.19	−0.01 (−0.06, 0.04)	0.78	0.03 (−0.03, 0.08)	0.32
**Maternal sFlt-1 concentrations**								
Basic model^b^	−0.04 (-0.08, -0.02) ^a^	0.01^a^	−0.02 (−0.05, 0.01)	0.26	0.02 (−0.02, -0.05)	0.29	−0.01 (−0.05, 0.02)	0.53
Confounder model^c^	−0.02 (−0.06, 0.01)	0.20	−0.01 (−0.05, 0.02)	0.51	0.01 (−0.02, 0.05)	0.49	−0.02 (−0.05, 0.02)	0.33
**Maternal sFlt-1/PlGF ratio**								
Basic model^b^	−0.05 (−0.09, -0.01) ^a^	0.01^a^	−0.05 (−0.09, -0.01) ^a^	0.01^a^	0.03 (−0.02, 0.07)	0.24	−0.01 (−0.06, 0.03)	0.53
Confounder model^c^	−0.02 (−0.06, 0.02)	0.43	−0.03 (−0.07, 0.01)	0.15	0.00 (−0.02, 0.04)	0.34	−0.03 (−0.08, 0.01)	0.15
**Second trimester**								
**Maternal PlGF concentrations**								
Basic model^b^	−0.01 (−0.05, 0.02)	0.40	−0.01 (−0.04, 0.03)	0.65	0.03 (−0.01, 0.06)	0.12	0.01 (−0.02, 0.05)	0.55
Confounder model^c^	−0.01 (−0.04, 0.02)	0.57	−0.02 (−0.05, 0.02)	0.39	0.02 (−0.01, 0.06)	0.19	0.01 (−0.03, 0.04)	0.67
**Maternal sFlt-1 concentrations**								
Basic model^b^	−0.04 (−0.07, -0.01) ^a^	0.02^a^	−0.03 (-0.06, 0.01)	0.10	0.02 (−0.01, 0.05)	0.23	−0.02 (−0.06, 0.01)	0.14
Confounder model^c^	−0.02 (−0.05, 0.01)	0.19	−0.02 (−0.06, 0.01)	0.14	−0.00 (−0.03, 0.03)	0.33	−0.03 (−0.06, 0.00)	0.08
**Maternal sFlt-1/PlGF ratio**								
Basic model^b^	−0.02 (−0.05, 0.01)	0.15	−0.02 (−0.05, 0.01)	0.26	−0.00 (−0.03, 0.03)	0.97	−0.03 (−0.06, 0.01)	0.10
Confounder model^c^	−0.01 (−0.04, 0.02)	0.47	−0.01 (−0.04, 0.02)	0.50	−0.00 (−0.03, 0.03)	0.95	−0.03 (−0.06, 0.00)	0.08

Values represent regression coefficients (95% confidence interval) and corresponding P-value from linear regression models that reflect differences in childhood vascular outcomes per 1 SDS increase in maternal PlGF and sFlt-1 concentrations or sFlt-1/PlGF ratio. SDS: standard deviation score.

a *p* < 0.05.

b Basic model, adjusted for gestational age at blood sampling, child’s age and sex.

c Confounder model, basic model additionally adjusted for age mother at intake, prepregnancy BMI, education, ethnicity, smoking, alcohol use and folic acid intake.

**Table 3:** Associations of maternal first and second trimester PlGF and sFlt-1 concentrations with childhood vascular outcomes at 9 years among children born SGA (n = 455), AGA (n = 3647) and LGA (n=455).

	Difference In childhood systolic blood pressure SDS (95%CI)						Difference In childhood diastolic blood pressure SDS (95%CI)						Difference In childhood Intlma-medla thickness (cIMT) SDS (95%CI)						Difference In childhood distensibility SDS (95%CI)					
Maternal angiogenesis factors	Children born SGA	*p*-value	Children born AGA	*p*-value	Children born LGA	*p*-value	Children born SGA	*p*-value	Children born AGA	*p*-value	Children born LGA	*p*-value	Children born SGA	*p*-value	Children born AGA	*p*-value	Children born LGA	*p*-value	Children born SGA	*p*-value	Children born AGA	*p*-value	Children born LGA	*p*-value
**First trimester**																								
Maternal PlGF concentrations	0.14 (−0.01, 0.29)	0.08	-0.03 (-0.09, 0.02)	0.22	-0.03 (-0.19, 0.14)	0.76	0.06 (-0.09, 0.21)	0.46	0.03 (−0.03, 0.09)	0.30	0.09 (-0.08, 0.25)	0.32	-0.12 (−0.29, 0.06)	0.19	0.00 (-0.06, 0.06)	0.99	-0.05 (-0.21, 0.12)	0.59	-0.13 (-0.29, 0.03)	0.11	0.04 (-0.02, 0.10)	0.17	0.13 (-0.06, 0.32)	0.19
Maternal sFlt-1 concentrations	0.04 (−0.07, 0.15)	0.50	-0.04 (−0.07, 0.00)	0.05	0.06 (-0.06, 0.18)	0.35	0.01 (-0.09, 0.12)	0.80	-0.02 (−0.06, 0.02)	0.35	0.03 (−0.10, 0.15)	0.64	-0.05 (-0.17, 0.07)	0.44	0.00 (-0.04, 0.04)	0.85	0.12 (0.00, 0.24)^a^	0.05	-0.03 (-0.14, 0.08)	0.63	-0.01 (-0.05, 0.03)	0.65	-0.07 (-0.21, 0.07)	0.33
Maternal sFlt-1/PlGF ratio	-0.04 (−0.16, 0.08)	0.50	-0.02 (-0.07, 0.02)	0.35	0.07(-0.06, 0.21)	0.30	-0.02 (-0.13, 0.10)	0.76	-0.04 (-0.21, 0.29)	0.11	-0.01 (-0.15, 0.14)	0.92	0.02 (-0.11, 0.15)	0.81	0.01 (-0.04, 0.06)	0.75	0.14 (0.00, 0.29)	0.05	0.04 (-0.08, 0.15)	0.56	-0.03 (-0.08, 0.02)	0.23	-0.14 (-0.30, 0.02)	0.10
**Second trimester**																								
Maternal PlGF concentrations	0.02 (−0.07, 0.11)	0.67	-0.02 (-0.05, 0.02)	0.40	-0.03 (−0.13, 0.08)	0.65	-0.05 (-0.14, 0.04)	0.28	-0.01 (-0.05, 0.03)	0.67	0.04 (-0.07, 0.15)	0.51	-0.09 (-0.19, 0.01)	0.06	0.04 (−0.01, 0.08)	0.09	0.06 (-0.05, 0.17)	0.27	-0.04 (-0.13, 0.06)	0.45	0.02 (-0.03, 0.06)	0.49	0.07 (-0.05, 0.20)	0.26
Maternal sFlt-1 concentrations	-0.01 (-0.10, 0.08)	0.87	-0.02 (-0.06, 0.01)	0.21	-0.02 (−0.12, 0.08)	0.65	-0.02 (0-1, 0.07)	0.71	-0.03 (−0.06, 0.01)	0.16	-0.01 (-0.11, 0.10)	0.89	-0.07 (-0.16, 0.03)	0.17	0.02 (-0.01, 0.06)	0.19	0.05 (-0.05, 0.15)	0.35	0.00 (-0.09, 0.09)	0.98	-0.03 (-0.06, 0.01)	0.16	-0.08 (-0.19, 0.04)	0.18
Maternal sFlt-1/PlGF ratio	-0.02 (-0.11, 0.07)	0.65	-0.01 (-0.05, 0.03)	0.61	0.00 (-0.10, 0.10)	0.95	0.02 (-0.07, 0.11)	0.64	-0.02 (-0.05, 0.02)	0.36	-0.03 (-0.13, 0.07)	0.58	0.01 (-0.09, 0.11)	0.88	0.00 (-0.04, 0.04)	0.99	0.00 (-0.10, 0.10)	0.95	0.03 (-0.07, 0.12)	0.57	-0.03 (-0.07, 0.01)	0.09	-0.11 (-0.22, 0.01)	0.06

Values represent regression coefficients (95% confidence interval) and corresponding *p*-value from linear regression models that reflect differences in childhood vascular outcomes per 1 SDS increase in maternal PlGF and sFlt-1 concentrations or sFlt-1/PlGF ratio, analyzed for children born SGA, AGA and LGA separately. Models were adjusted for gestational age at intake, gestational age at blood sampling, educational level, ethnicity, parity, prepregnancy BMI, blood pressure, smoking, alcohol consumption, folic acid supplement use and child’s age and sex. SDS: standard deviation score SGA: small for gestational age, AGA: appropriate for gestational age LGA: large for gestational age.

a *p*<0.05.

**Table 4:** Associations of maternal first and second trimester PlGF and sFlt-1 concentrations with childhood vascular outcomes at 9 years among children born preterm (n = 206) and term (n = 4359).

	Difference in systolic childhood blood pressure SDS (95%CI)				Difference in diastolic childhood blood pressure SDS (95%CI)				Difference in childhood intima-media thickness (cIMT) SDS (95%CI)				Difference in childhood distensibility SDS (95%CI)			
Maternal angiogenesis factors	Children born preterm	*p*-value	Children born term	*p*-value	Children born preterm	*p*-value	Children born term	*p*-value	Children bornpreterm	*p*-value	Children born term	*p*-value	Children born preterm	*p*-value	Children born term	*p*-value
**First trimester**																
Maternal PlGF concentrations	−0.07 (−0.32, 0.19)	0.62	−0.01 (−0.06, 0.04)	0.67	−0.14 (−0.40, 0.12)	0.29	0.04− (−0.01, 0.10)	0.12	−0.12 (−0.43, 0.19)	0.44	−0.01 (−0.06, 0.05)	0.78	−0.19 (−0.48, 0.20)	0.20	0.04 (−0.02, 0.09)	0.56
Maternal sFlt−1 concentrations	0.10 (−0.08, 0.28)	0.28	−0.03 (−0.06, 0.01)	0.11	−0.01 (−0.19, 0.17)	0.93	−0.01 (−0.05, 0.02)	0.56	−0.06 (−0.27, 0.15)	0.57	0.02 (−0.02, 0.05)	0.39	−0.12 (−0.31, 0.08)	0.24	−0.01 (−0.05, 0.03)	0.58
Maternal sFlt−1/PlGF ratio	0.14 (−0.07, 0.34)	0.19	−0.02 (−0.07, 0.02)	0.25	0.08 (−0.13, 0.29)	0.47	−0.03 (−0.08, 0.01)	0.12	0.01 (−0.23, 0.26	0.91	0.02 (−0.02, 0.07)	0.29	−0.02 (−0.25, 0.22)	0.90	−0.03 (−0.07, 0.02)	0.23
**Second trimester**																
Maternal PlGF concentrations	−0.09 (−0.23, 0.05)	0.20	−0.00 (−0.04, 0.03)	0.80	−0.16 (−0.30, −0.03)^a^	0.02^a^	−0.01 (−0.04, 0.03)	0.80	−0.04 (−0.19, 0.12)	0.65	0.03 (−0.01, 0.06)	0.13	0.05 (−0.10, 0.20)	0.50	0.01 (−0.03, 0.04)	0.78
Maternal sFlt−1 concentrations	0.12 (−0.04, 028)	0.15	−0.03 (−0.06, 0.00)	0.09	0.12 (−0.04, 0.28)	0.14	−0.03 (−0.06, 0.00)	0.06	0.03 (−0.15, 0.21)	0.75	0.02 (−0.02, 0.05)	0.33	0.01 (−0.16, 0.18)	0.91	−0.03 (−0.07, 0.00)	0.05
Maternal sFlt−1/PlGF ratio	0.14 (−0.00, 0.27)	0.05	−0.02 (−0.05, 0.01)	0.20	0.19 (0.06, 0.16)^a^	0.01^a^	−0.02 (−0.06, 0.01)	0.15	0.04 (−0.11, 0.20)	0.59	−0.00 (−0.04, 0.02)	0.87	−0.03 (−0.18, 012)	0.69	−0.03 (−0.07, 0.00)	0.06

a *p*<0.05. Values represent regression coefficients (95% confidence interval) and corresponding *p*-value from linear regression models that reflect differences in childhood vascular outcomes per 1 SDS increase in maternal PlGF and sFlt-1 concentrations or sFlt-1/PlGF ratio, analyzed for children born preterm (<37 weeks) and term (>37 weeks) separately. Models were adjusted for gestational age at intake, gestational age at blood sampling, educational level, ethnicity, parity, prepregnancy BMI, blood pressure, smoking, alcohol consumption, folic acid supplement use and child’s age and sex. SDS: standard deviation score.
