# Supplementary material for: Associations of maternal angiogenic factors during pregnancy with childhood carotid intima-media thickness and blood pressure
Source: Atherosclerosis. Author manuscript; Available in PMC 2022 Oct 27. (PMC7613754; doi:10.1016/j.atherosclerosis.2021.11.005)
Supplement: Supplementary Material [file EMS156026-supplement-Supplementary_Material.docx]

Supplementary material

**Associations of maternal angiogenic factors during pregnancy with childhood carotid intima-media thickness and blood pressure**

*Running title: Maternal PlGF and sFlt-1 concentrations and childhood vascular outcomes*

Meddy N. Bongers-Karmaoui MD^1,2^, Vincent W.V. Jaddoe MD PhD^1,2^, Romy Gaillard MD PhD^1,2^

1. The Generation R Study Group, Erasmus University Medical Center, Rotterdam, the Netherlands.
2. Department of Pediatrics, Sophia Children's Hospital, Erasmus University Medical Center, Rotterdam, the Netherlands.

Corresponding author

Romy Gaillard, The Generation R Study Group, Erasmus University Medical Center, PO Box 2040, 3000 CA Rotterdam, The Netherlands (r.gaillard@erasmusmc.nl). Telephone number: 0031 10 704 3405

| **Page** |  | |
| --- | --- | --- |
| 1 | **Figure S1.** | Directed acyclic graph to identify potential confounders and mediators. |
| 2 | **Table S1.** | Non-response analyses for mothers with vs. without serum PLGF and sFlt-2 measurements in pregnancy. |
| 3 | **Table S2.** | Non-response analyses for mothers and their offspring with vs. without vascular measurements at 9 years. |
| 4 | **Table S3.** | Characteristics of mothers and their children in the Generation R study for children born SGA, AGA and LGA. |
| 5 | **Table S4.** | Characteristics of mothers and their children in the Generation R study for children born preterm and term. |
| 6 | **Table S5.** | Characteristics of mothers and their children in the Generation R study for girls and boys. |
| 7 | **Table S6.** | P-values for testing interaction terms. |
| 8 | **Figure S2.** | Regression analysis with maternal first trimester PlGF concentrations in quintiles. |
| 9 | **Figure S3.** | Regression analysis with maternal first trimester sFlt-1 concentrations in quintiles. |
| 10 | **Figure S4.** | Regression analysis with maternal first trimester PlGF/sFlt-1 ratio in quintiles. |
| 11 | **Figure S5.** | Regression analysis with maternal second trimester PlGF concentrations in quintiles. |
| 12 | **Figure S6.** | Regression analysis with maternal second trimester sFlt-1 concentrations in quintiles. |
| 13 | **Figure S7.** | Regression analysis with maternal second trimester PlGF/sFlt-1 ratio in quintiles. |

**Figure S1** Directed acyclic graph to identify potential confounders and mediators


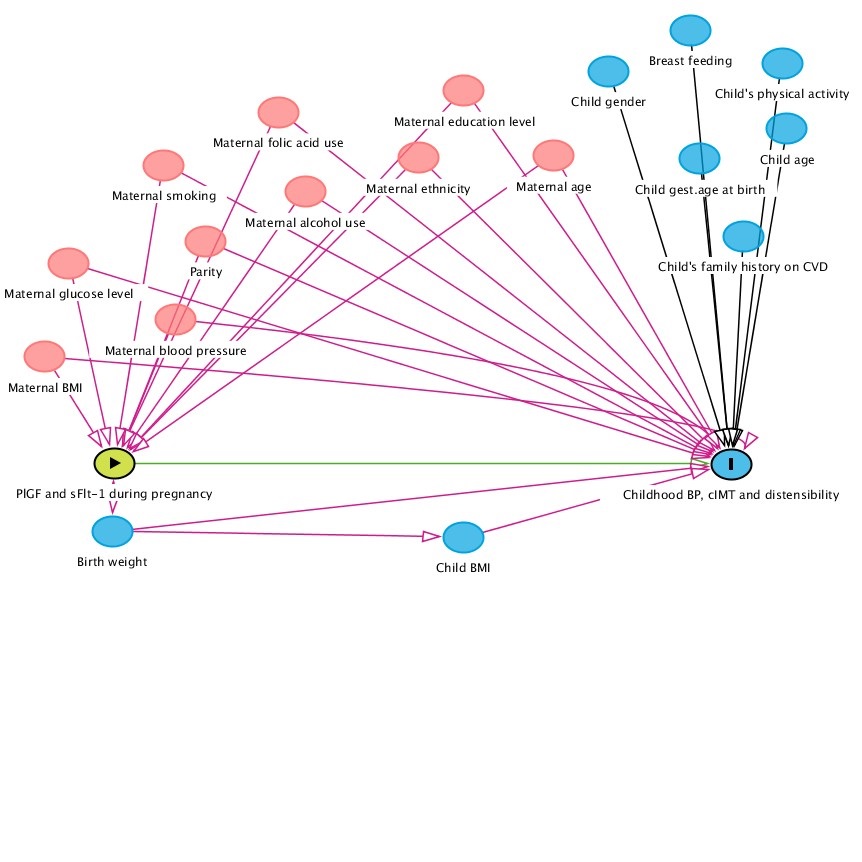


**Table S1** Non-response analysis for women with and without PlGF and sFlt-1 measurements in pregnancy.

|  | PlGF and sFlt-1  measurements  in pregnancy  N= 8010 | No PlGF and sFlt-1  Measurements  in pregnancy  N= 623 |
| --- | --- | --- |
| Maternal Characteristics |  |  |
| Age at enrolment, mean (SD), years | 29.6 (5.2) | 28.9 (6.1) |
| Gestational age at intake, median (95%), weeks | 14.2 (10.2-22.8) | 21.5 (11.0-34.7) |
| Prepregnancy BMI, median (95%), kg/m^2^ | 22.6 (18.0-35.2) | 22.7 (17.5-33.5) |
| Parity, No. nulliparous (%) | 4419 (55.7) | 327 (54.9) |
| Ethnicity, no. Dutch or European (%) | 4387 (57.6) | 251 (46.1) |
| Education level, n high (%) | 3102 (42.3) | 184 (35.9) |
| Smoking during pregnancy, n yes (%) | 1317 (18.7) | 89 (18.2) |
| Folic acid supplement use, n yes (%) | 2379 (40.0) | 130 (31.9) |
| Pregnancy induced hypertension, n yes (%) | 292 (3.7) | 19 (3.2) |
|  | |  |
| Child Characteristics |  |  |
| Age, mean (SD), years | 9.8 (0.4) | 9.9 (0.5) |
| Gender, n female (%) | 3979 (49.7) | 298 (48.0) |
| Birth weight, mean (SD), grams | 3412.6 (635.0) | 3385.8 (551.0) |
| Gestational age at birth, median (95%),  weeks | 40.1 (35.4-42.3) | 40.0 (35.5-42.4) |
| BMI, median (95%), kg/m^2^ | 17.0 (14.0-25.0) | 17.6 (14.2-24.9) |
| Systolic blood pressure, mean (SD),  mmHg | 103.1 (7.9) | 103.3 (8.2) |
| Diastolic blood pressure, mean (SD),  mmHg | 58.6 (6.4) | 58.5 (6.6) |
| Carotid intima-media thickness, median  (95%), mm | 0.46 (0.37-0.54) | 0.46 (0.36-0.56) |
| Carotid distensibility, median (95%),  10-3*kPa-1 | 55.8 (37.1-85.5) | 57.2 (34.2-90.2) |

For normal distributed data, the mean with standard deviation is stated. For non-normally distributed data, the median with 95% range is stated.

**Table S2** Non-response analysis for women and their children with and without blood pressure and vascular ultrasonography measurements at 9 years.

|  | Blood pressure  and vascular  ultrasonography measurements  N= 4881 | No blood pressure  and vascular  ultrasonography measurements  N= 3752 |
| --- | --- | --- |
| Maternal Characteristics |  |  |
| Age at enrolment, mean (SD), years | 30.7 (4.9) | 28.2 (5.5) |
| Gestational age at intake, median (95%), weeks | 14.2 (10.4-25.3) | 14.8 (10.2-30.1) |
| Prepregnancy BMI, median (95%), kg/m^2^ | 22.6 (18.1-34.6) | 22.7 (17.7-35.5) |
| Parity, No. nulliparous (%) | 2830 (58.3) | 1916 (52.2) |
| Ethnicity, no. Dutch or European (%) | 3089 (64.5) | 1549 (45.9) |
| Education level, n high (%) | 2308 (49.9) | 978 (30.4) |
| Smoking during pregnancy, n yes (%) | 670 (15.4) | 736 (23.2) |
| Folic acid supplement use, n yes (%) | 1719 (45.9) | 803 (30.4 ) |
| Pregnancy induced hypertension, n yes (%) | 189 (4.0) | 122 (3.4) |
| First trimester PlGF, median (95%), pg/ml | 41.3 (14.5-191.0) | 45.9 (14.7-204.8) |
| First trimester sFlt-1, median (95%), pg/ml | 5.0 (1.9-13.6) | 5.2 (1.9-15.3) |
| Second trimester PlGF, median (95%), pg/ml | 196.2 (73.9-602.7) | 210.3 (71.7-660.5) |
| Second trimester sFlt-1, median (95%), pg/ml | 4.9 (1.5-16.7) | 5.0 (1.6-18.4) |
|  | |  |
| Child Characteristics |  |  |
| Age, mean (SD), years | 9.8 (0.3) | 10.0 (0.8) |
| Gender, n female (%) | 2480 (50.8) | 1797 (47.9) |
| Birth weight, mean (SD), grams | 3434.5 (552.6) | 3379.2 (571.0) |
| Gestational age at birth, median (95%),  weeks | 40.1 (35.9-42.3) | 40.0 (35.0-42.3) |
| BMI, median (95%), kg/m^2^ | 17.0 (14.0-24.9) | 16.9 (13.7-25.1) |

For normal distributed data, the mean with standard deviation is stated. For non-normally distributed data, the median with 95% range is stated.

**Table S3.** Characteristics of mothers and their children in the Generation R study for children born preterm and term.

|  | Preterm born children  N=206 | Term born children  N=4359 |
| --- | --- | --- |
| Maternal Characteristics | |  |
| First trimester PlGF, median (95%), pg/ml | 35.8 (10.8-195.4) | 41.5 (14.8-191.1) |
| Second trimester PlGF, median (95%), pg/ml | 200.2 (37.2-635.1) | 195.8 (75.7-594.4) |
| First trimester sFlt-1, median (95%), pg/ml | 4.6 (1.5-12.0) | 5.0 (1.9-13.6) |
| Second trimester sFlt-1, median (95%), pg/ml | 5.0 (1.6-18.3) | 4.9 (1.5-16.6) |
| First trimester maternal sFlt-1/PlGF ratio, median (95%), | 0.12 (0.02-0.57) | 0.12 (0.02-0.42) |
| Second trimester maternal sFlt-1/PlGF ratio, median (95%), | 0.02 (0.01-0.23) | 0.03 (0.02-0.42) |
| Child Characteristics | | |
| Start of birth, no. spontaneously (%) | 145 (88.4) | 3429 (86.4) |
| Birth weight, mean (SD), grams | 2318.2 (615.2)* | 3490.8 (493.0)* |
| Gestational age at birth, median (95%), weeks | 35.6 (27.4-36.9)* | 40.3 (37.4-42.4)* |
| Systolic blood pressure,  mean (SD), mmHg | 103.9 (8.6) | 103.1 (7.9) |
| Diastolic blood pressure,  mean (SD), mmHg | 59.2 (6.7) | 58.6 (6.4) |
| Carotid intima-media  thickness, median (95%), mm | 0.45 (0.35-0.55) | 0.46 (0.37-0.54) |
| Carotid distensibility, median  (95%), 10-3*kPa-1 | 56.0 (36.2-94.7) | 55.8 (37.1-85.2) |

Values are observed data and represent means (SD), medians (95% range) or numbers of subjects (valid %). Differences in characteristics between the preterm and term born children were evaluated using Student's t-tests for independent samples and Pearson Chi-Square for categorical variables. *P<0.05

|  | Children born  SGA N=455 | Children born  AGA N=4359 | | Children born  LGA N=455 |
| --- | --- | --- | --- | --- |
| Maternal Characteristics | | |  |  |
| First trimester PlGF, median (95%), pg/ml | 40.6 (13.2-189.4) | 41.7 (14.8-192.2) | | 40.9 (16.6-176.4) |
| Second trimester PlGF, median (95%), pg/ml | 194.0 (41.4-630.1) | 196.1 (77.0-600.1) | | 197.0 (76.3-599.5) |
| First trimester sFlt-1, median (95%), pg/ml | 4.6 (1.8-13.3) | 5.0 (1.9-13.9) | | 5.1 (1.9-12.9) |
| Second trimester sFlt-1, median (95%), pg/ml | 4.6 (1.3-18.0) | 5.0 (1.6-16.9) | | 5.0 (1.5-15.5) |
| First trimester maternal sFlt-1/PlGF ratio, median (95%) | 0.11 (0.02-0.51) | 0.12 (0.02-0.42) | | 0.13 (0.02-0.41) |
| Second trimester maternal sFlt-1/PlGF ratio, median (95%) | 0.02 (0.00-0.18) | 0.03 (0.01-0.10) | | 0.02 (0.01-0.10) |
| Child Characteristics | | | | |
| Start of birth, no. spontaneously (%) | 321 (78.9)* | 2916 (88.1) | | 335 (81.5)* |
| Birth weight, mean (SD), grams | 2640.9 (363.6)* | 3432.4 (428.4) | | 4282.6 (363.2)* |
| Gestational age at birth, median (95%), weeks | 39.9 (35.3-42.1)* | 40.1 (35.9-42.4) | | 40.4 (36.3-42.4)* |
| Systolic blood pressure,  mean (SD), mmHg | 103.3 (8.3) | 103.1 (7.9) | | 103.1 (7.9) |
| Diastolic blood pressure,  mean (SD), mmHg | 59.1 (6.7) | 58.6 (6.4) | | 58.3 (6.4) |
| Carotid intima-media  thickness, median (95%), mm | 0.45 (0.35-0.54)* | 0.46 (0.37-0.54) | | 0.46 (0.38-0.54) |
| Carotid distensibility, median  (95%), 10-3*kPa-1 | 56.9 (37.8-84.9)* | 55.7 (37.4-85.9) | | 55.6 (34.6-82.7)* |

**Table S4.** Characteristics of mothers and their children in the Generation R study for children born SGA, AGA and LGA.

Values are observed data and represent means (SD) or medians (95% range).Differences in characteristics between SGA and AGA and between LGA and AGA born children were evaluated using one-way ANOVA analysis for independent samples and Pearson Chi-Square for categorical variables. SGA: small for gestational age, AGA: appropriate for gestational age LGA: large for gestational age *P<0.05

**Table S5.** Characteristics of mothers and their children in the Generation R study for girls and boys.

|  | Girls  N=2321 | Boys  N=2244 |  |
| --- | --- | --- | --- |
| Maternal Characteristics | |  |  |
| First trimester PlGF, median (95%), pg/ml | 41.7 (15.2-189.0) | 41.1 (13.9-193.3) |  |
| Second trimester PlGF, median (95%), pg/ml | 190.3 (71.6-582.0)* | 200.5 (75.4-612.6)* |  |
| First trimester sFlt-1, median (95%), pg/ml | 5.2 (2.0-14.4)* | 4.8 (1.8-13.0)* |  |
| Second trimester sFlt-1, median (95%), pg/ml | 5.1 (1.7-17.6)* | 4.7 (1.4-15.6)* |  |
| Child Characteristics | | |  |
| Start of birth, no. spontaneously (%) | 1814 (87.1) | 1760 (85.9) |  |
| Birth weight, mean (SD), grams | 3366.2 (542.4)* | 3512.9 (557.8)* |  |
| Gestational age at birth, median (95%), weeks | 40.1 (35.7-42.1)* | 40.3 (36.0-42.4)* |  |
| Systolic blood pressure,  mean (SD), mmHg | 103.6 (8.0)* | 102.7 (7.8)* |  |
| Diastolic blood pressure,  mean (SD), mmHg | 59.1 (6.2)* | 58.1 (6.5)* |  |
| Carotid intima-media  thickness, median (95%), mm | 0.45 (0.37-0.54)* | 0.46 (0.38-0.55)* |  |
| Carotid distensibility, median  (95%), 10-3*kPa-1 | 57.4 (38.4-86.9)* | 54.5 (36.3-83.4)* |  |

Values are observed data and represent means (SD) or medians (95% range). Differences in characteristics between girls and boys were evaluated using Student's t-tests for independent samples and Pearson Chi-Square for categorical variables. *P<0.05

**Table S6** P-values for interaction terms

|  | SBP | DBP | cIMT | DIS |
| --- | --- | --- | --- | --- |
| Interaction term |  |  |  |  |
| First trimester |  |  |  |  |
| PlGF*Gender | 0.21 | 0.97 | 0.24 | 0.70 |
| sFlt-1*Gender | 0.57 | 0.32 | 0.61 | 0.85 |
| sFlt-1/PlGF*Gender | 0.13 | 0.57 | 0.45 | 0.68 |
| PlGF*Gestational age adjusted birth weight | 0.42 | 0.45 | 0.95 | 0.04* |
| sFlt-1*Gestational age adjusted birth weight | 0.75 | 0.58 | 0.07 | 0.32 |
| sFlt-1/PlGF*Gestational age adjusted birth weight | 0.39 | 0.92 | 0.19 | 0.03* |
| PlGF*Gestational age at birth | 0.69 | 0.27 | 0.21 | 0.02* |
| sFlt-1*Gestational age at birth | 0.08 | 0.75 | 0.65 | 0.03* |
| sFlt-1/PlGF*Gestational age at birth | 0.26 | 0.42 | 0.35 | 0.75 |
| Second trimester |  |  |  |  |
| PlGF*Gender | 0.12 | 0.99 | 0.63 | 0.63 |
| sFlt-1*Gender | 0.41 | 0.77 | 0.78 | 0.39 |
| sFlt-1/PlGF*Gender | 0.06 | 0.85 | 0.56 | 0.31 |
| PlGF*Gestational age adjusted birth weight | 0.13 | 0.70 | 0.04* | 0.44 |
| sFlt-1*Gestational age adjusted birth weight | 0.69 | 0.92 | 0.05* | 0.17 |
| sFlt-1/PlGF*Gestational age adjusted birth weight | 0.47 | 0.83 | 0.86 | 0.10 |
| PlGF*Gestational age at birth | 0.92 | 0.11 | 0.24 | 0.52 |
| sFlt-1*Gestational age at birth | 0.03* | 0.05 | 0.81 | 0.39 |
| sFlt-1/PlGF*Gestational age at birth | 0.08 | 0.08 | 0.36 | 0.82 |

P-values for interaction terms in the association of PlGF and sFlt-1 concentrations and sFlt-1/PlGF ratio with vascular outcomes in childhood. SBP: systolic blood pressure, DBP: diastolic blood pressure, cIMT: carotid intima-media thickness, DIS: distensibility *p<0.05

**Figure S2** Regression analysis with maternal first trimester PlGF concentrations in quintiles


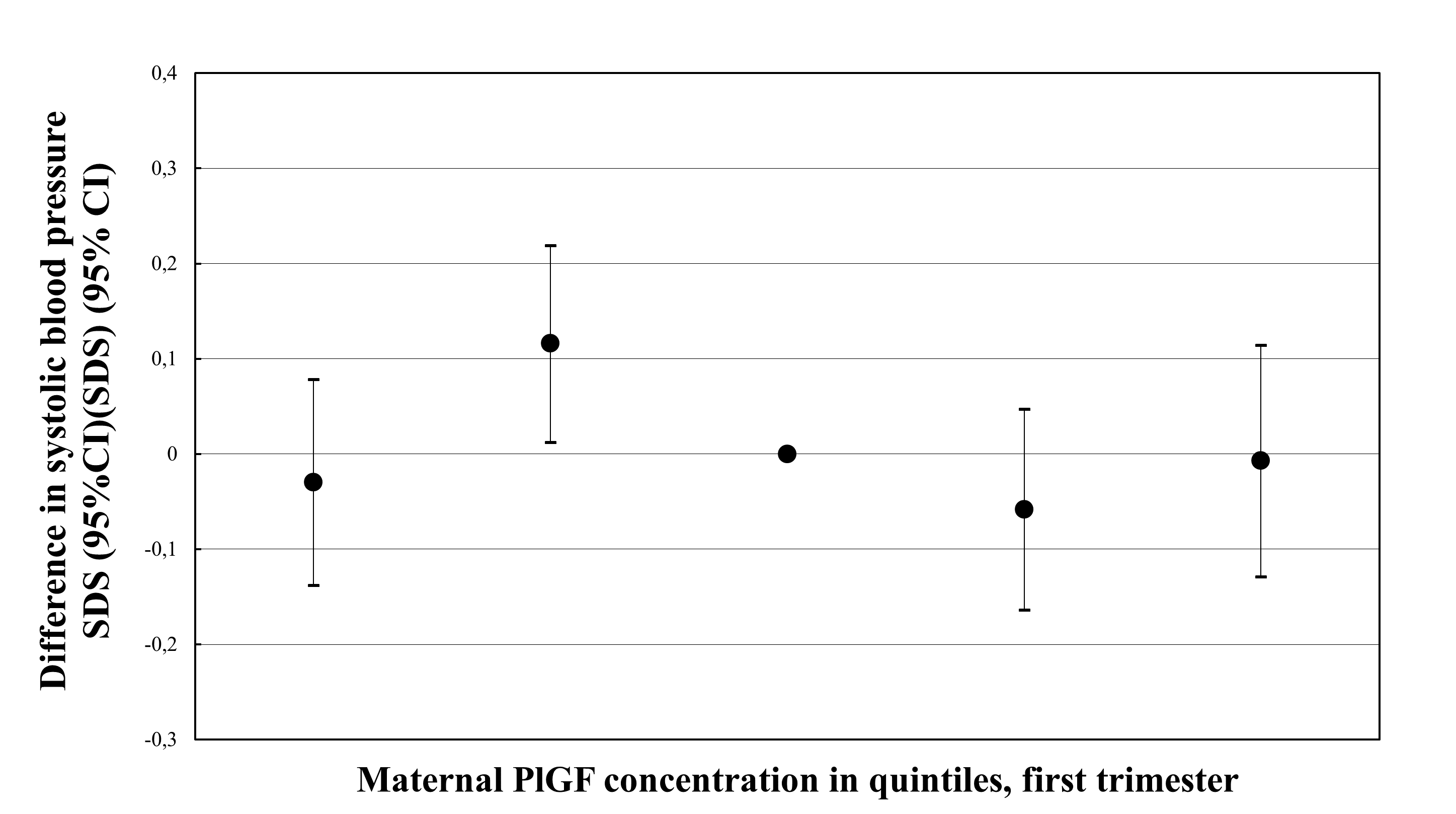

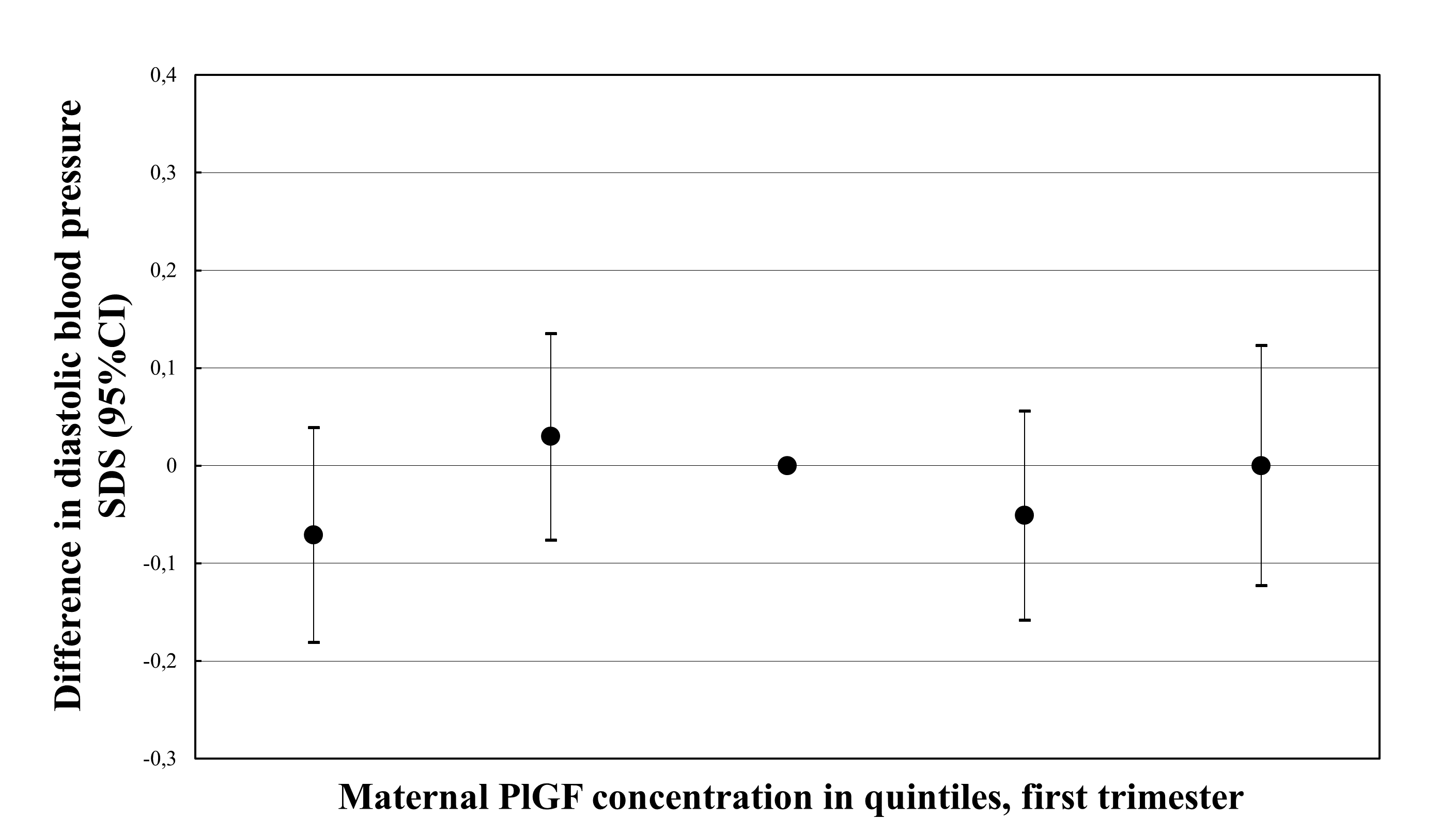


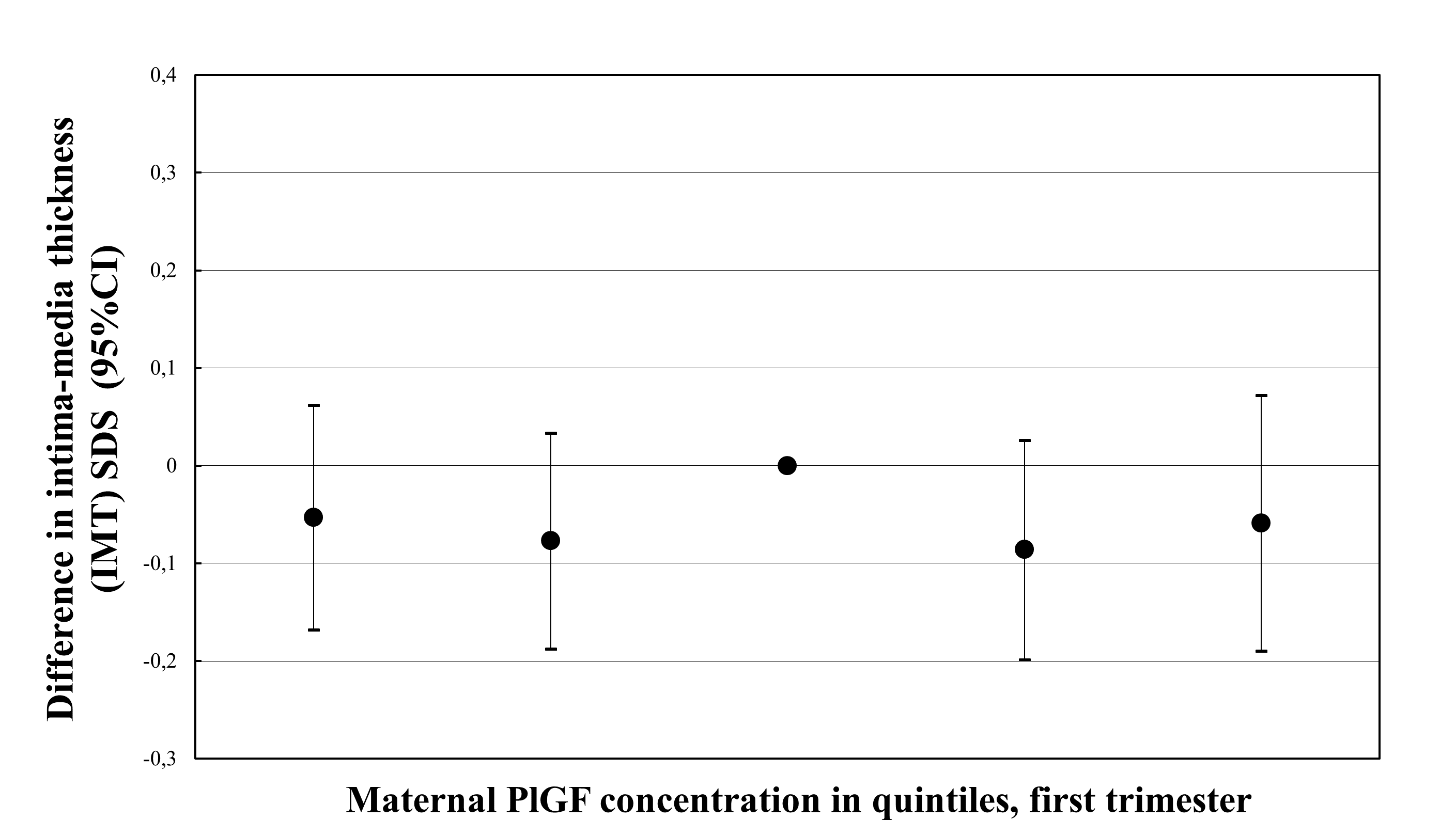

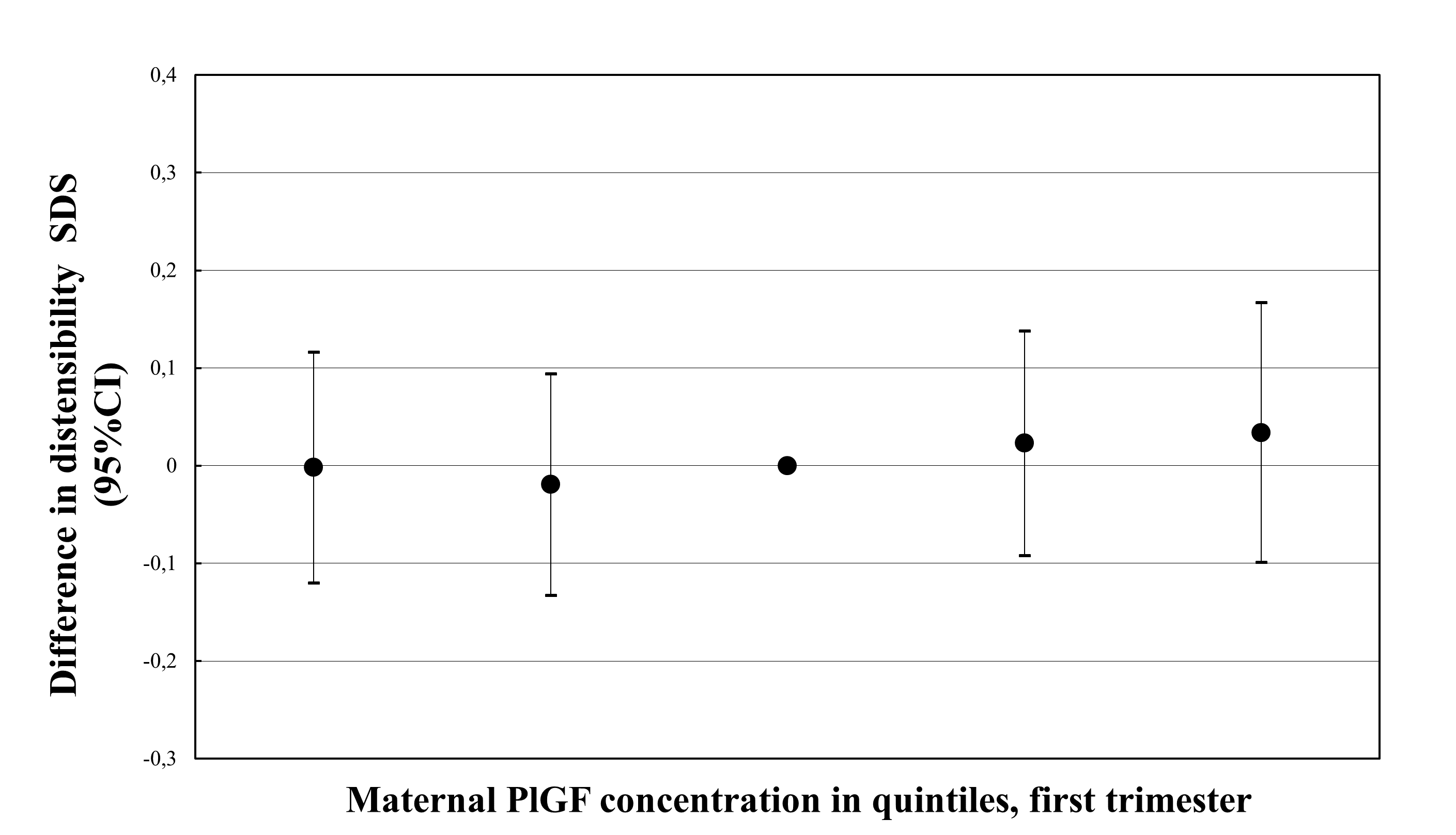


Regression analysis with childhood systolic blood pressure, diastolic blood pressure, carotid intima media thickness and carotid distensibility as dependent variables and maternal PlGF in first trimester as independent variable. Each point shows the strength of association (±95% CI) PlGF was divided in quintiles. The third quintile was the reference group. There was an adjustment for gestational age at intake, gestational age at blood sampling, educational level, ethnicity, parity, prepregnancy BMI, blood pressure, smoking, alcohol consumption, folic acid supplement use and child’s age and sex. 1): first trimester PlGF and childhood systolic blood pressure 2): first trimester PlGF and childhood diastolic blood pressure 3): first trimester PlGF and carotid intima media thickness 4): first trimester PlGF and carotid distensibility

**Figure S3** Regression analysis with maternal first trimester sFlt-1 concentrations in quintiles


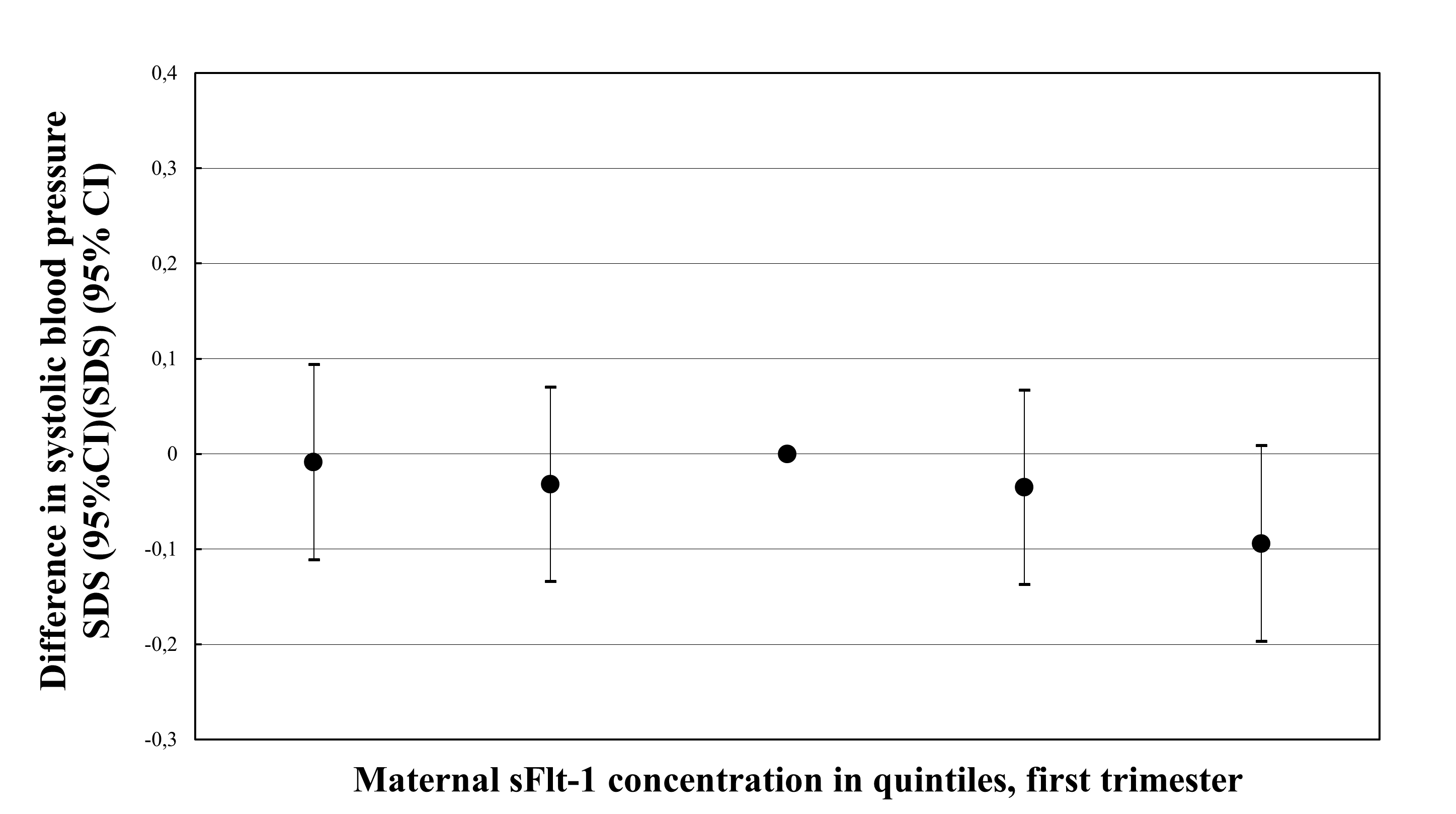

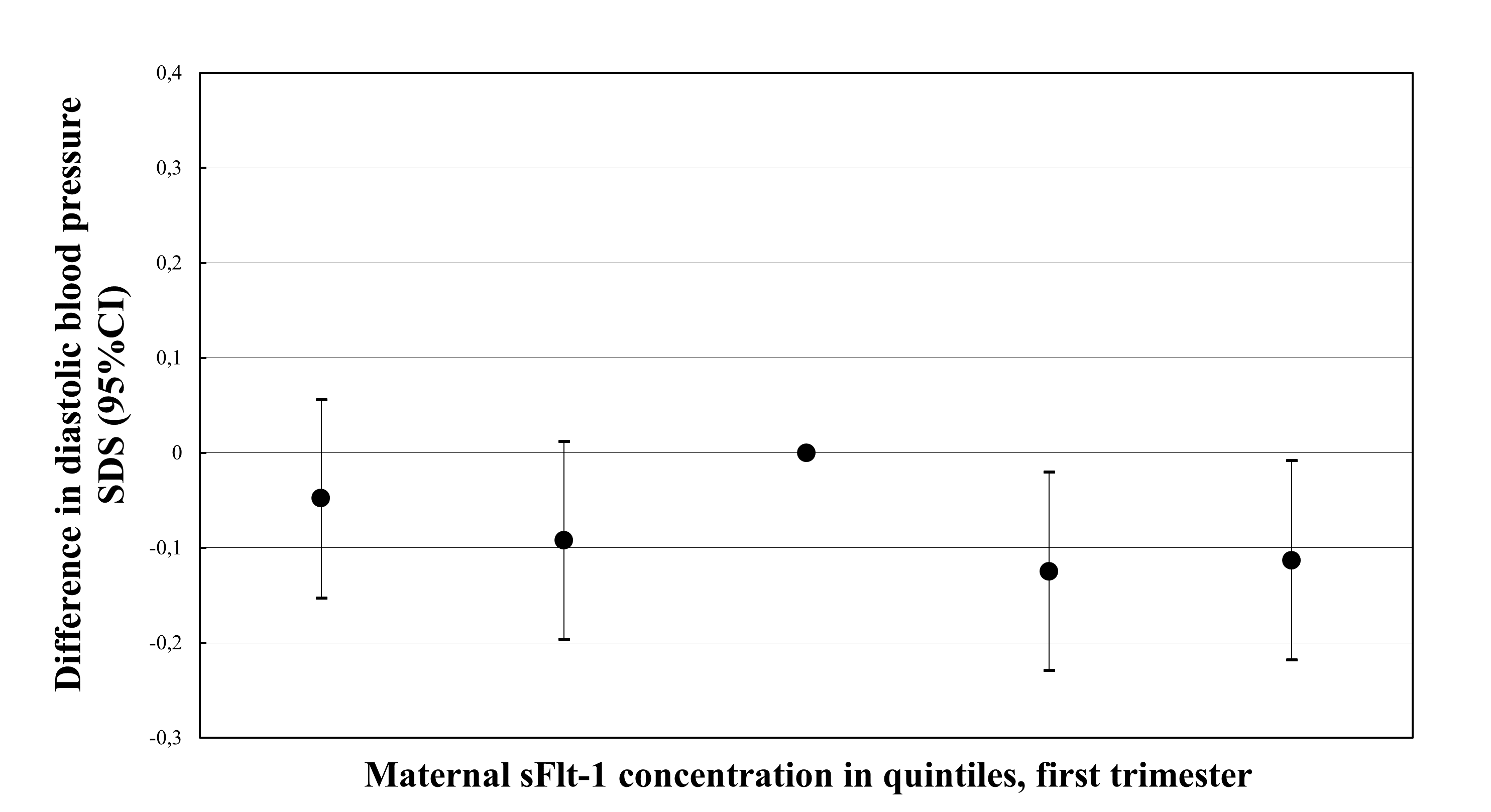


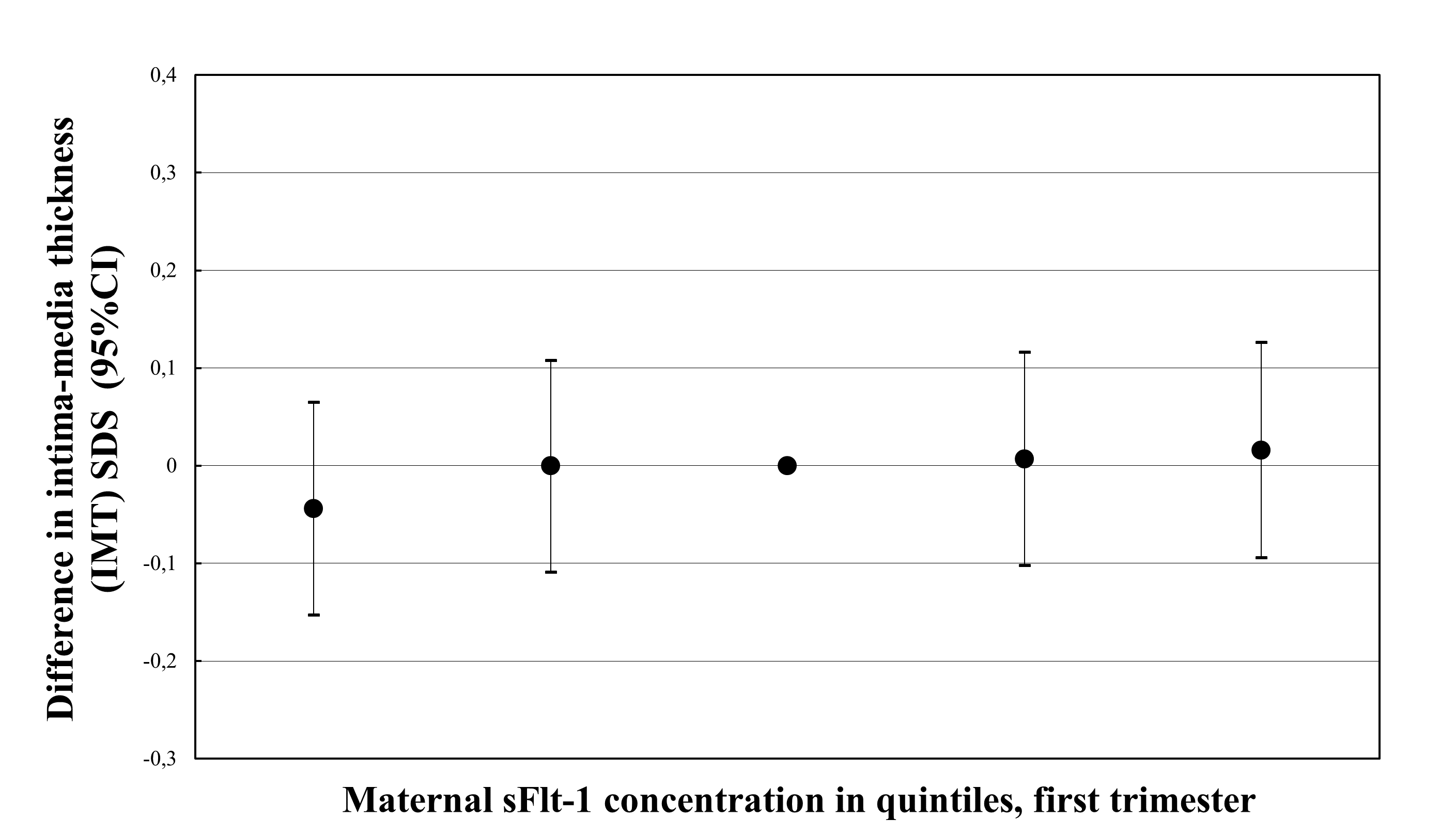

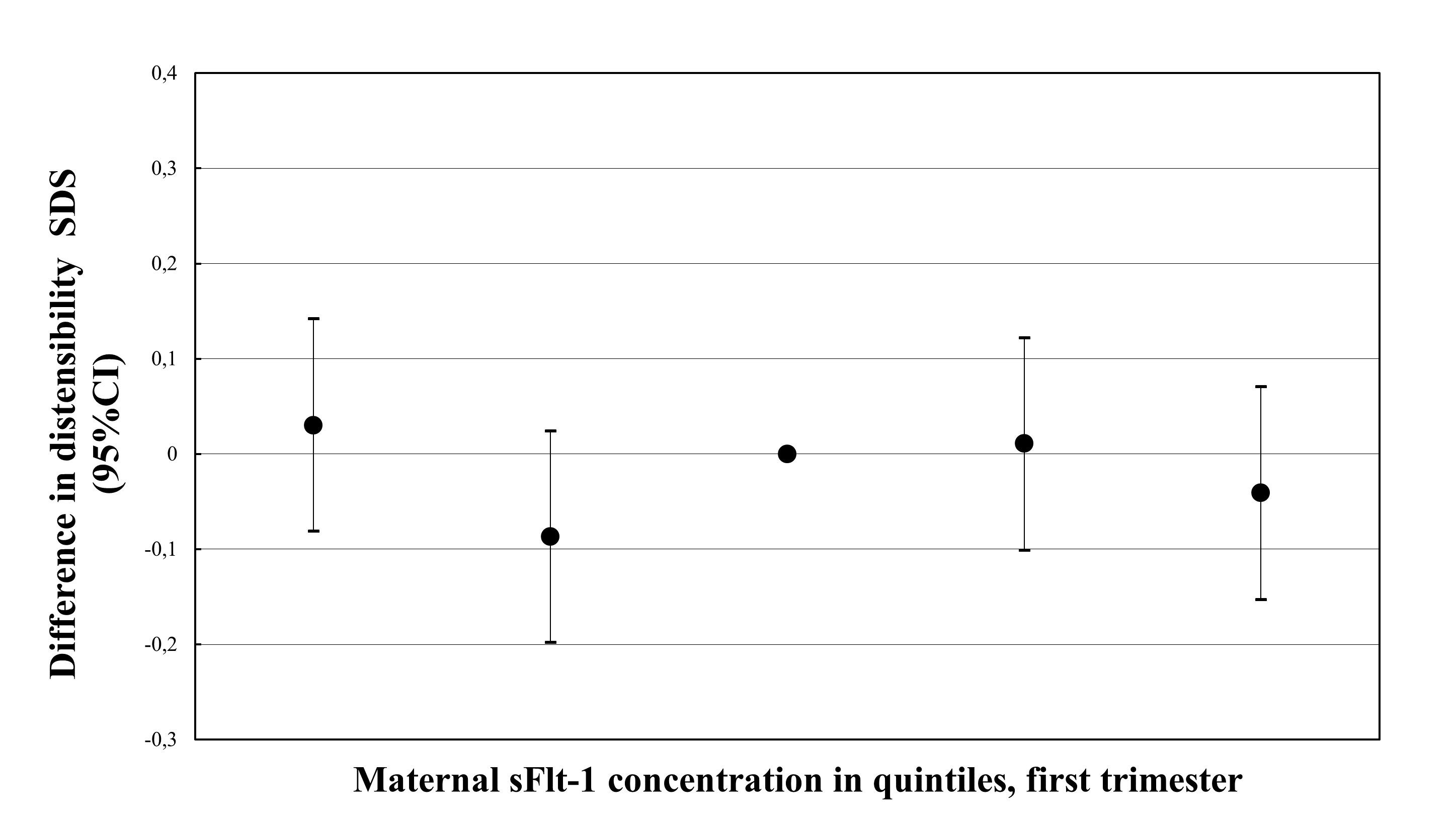


Regression analysis with childhood systolic blood pressure, diastolic blood pressure, carotid intima media thickness and carotid distensibility as dependent variables and maternal sFlt-1 in first trimester as independent variable. Each point shows the strength of association (±95% CI) sFlt-1 was divided in quintiles. The third quintile was the reference group. There was an adjustment for gestational age at intake, gestational age at blood sampling, educational level, ethnicity, parity, prepregnancy BMI, blood pressure, smoking, alcohol consumption, folic acid supplement use and child’s age and sex. 1): first trimester sFlt-1 and childhood systolic blood pressure 2): first trimester sFlt-1 and childhood diastolic blood pressure 3): first trimester sFlt-1 and carotid intima media thickness 4): first trimester sFlt-1 and carotid distensibility

**Figure S4** Regression analysis with maternal first trimester PlGF/sFlt1 ratio in quintiles


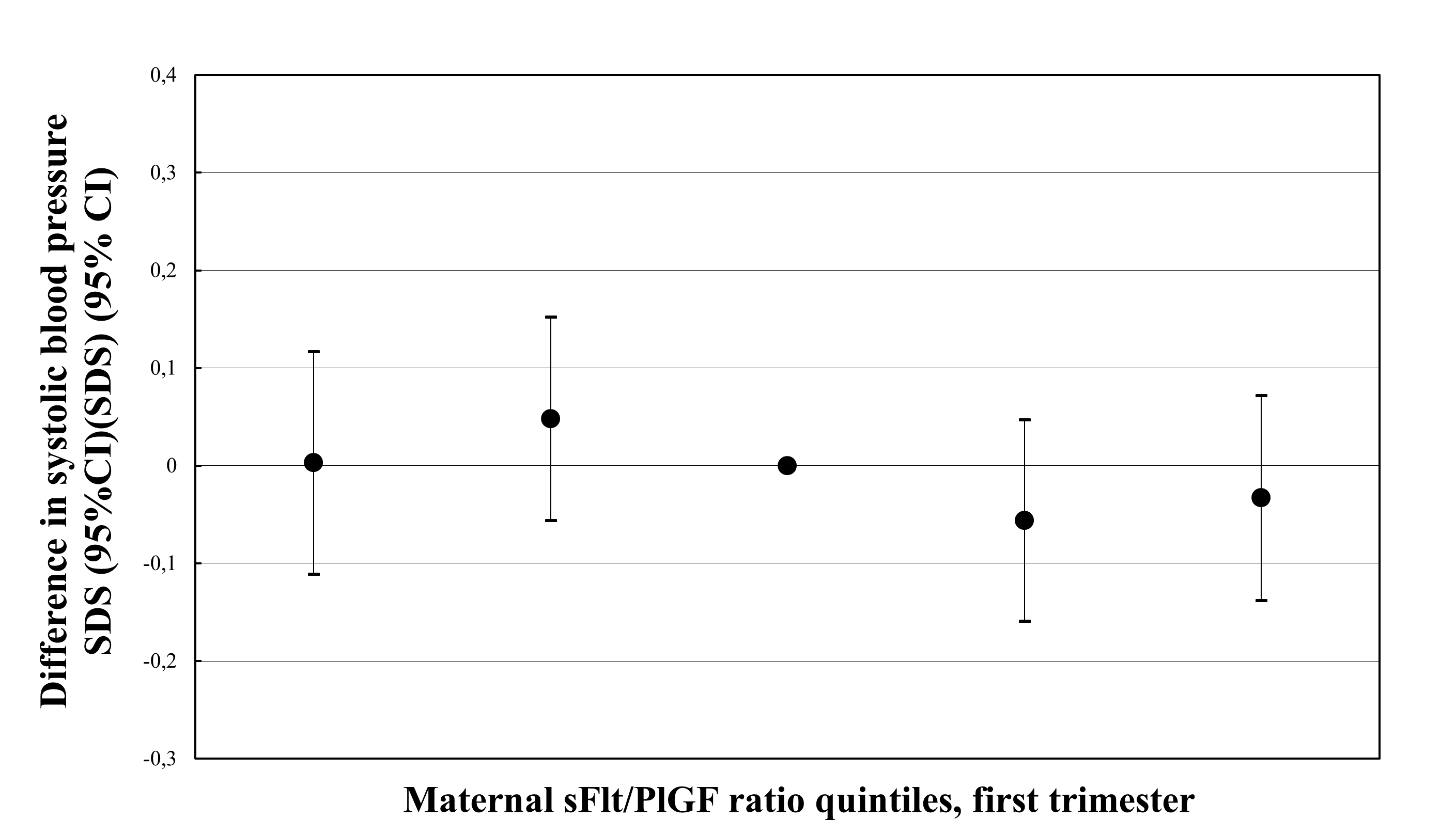

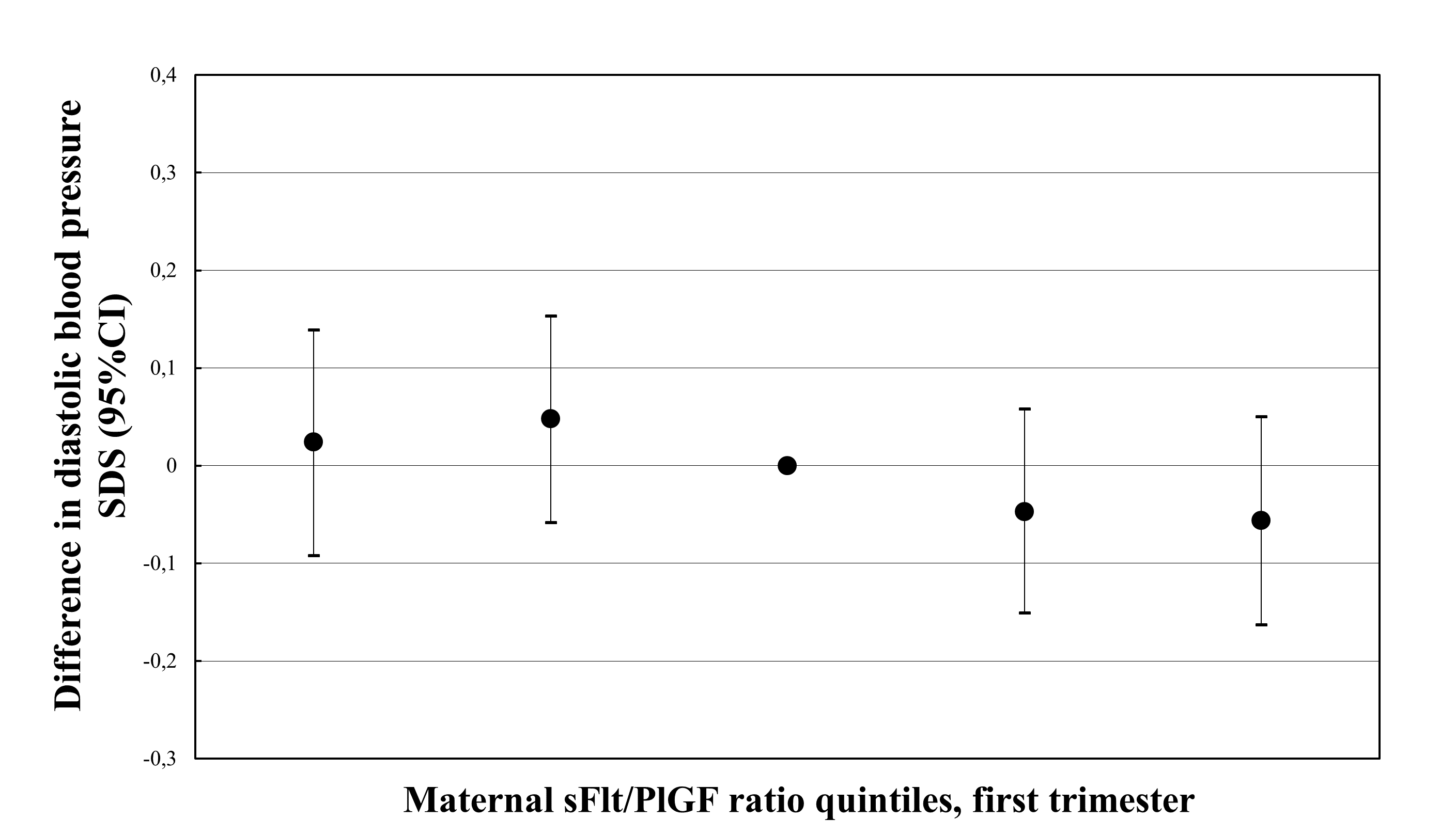


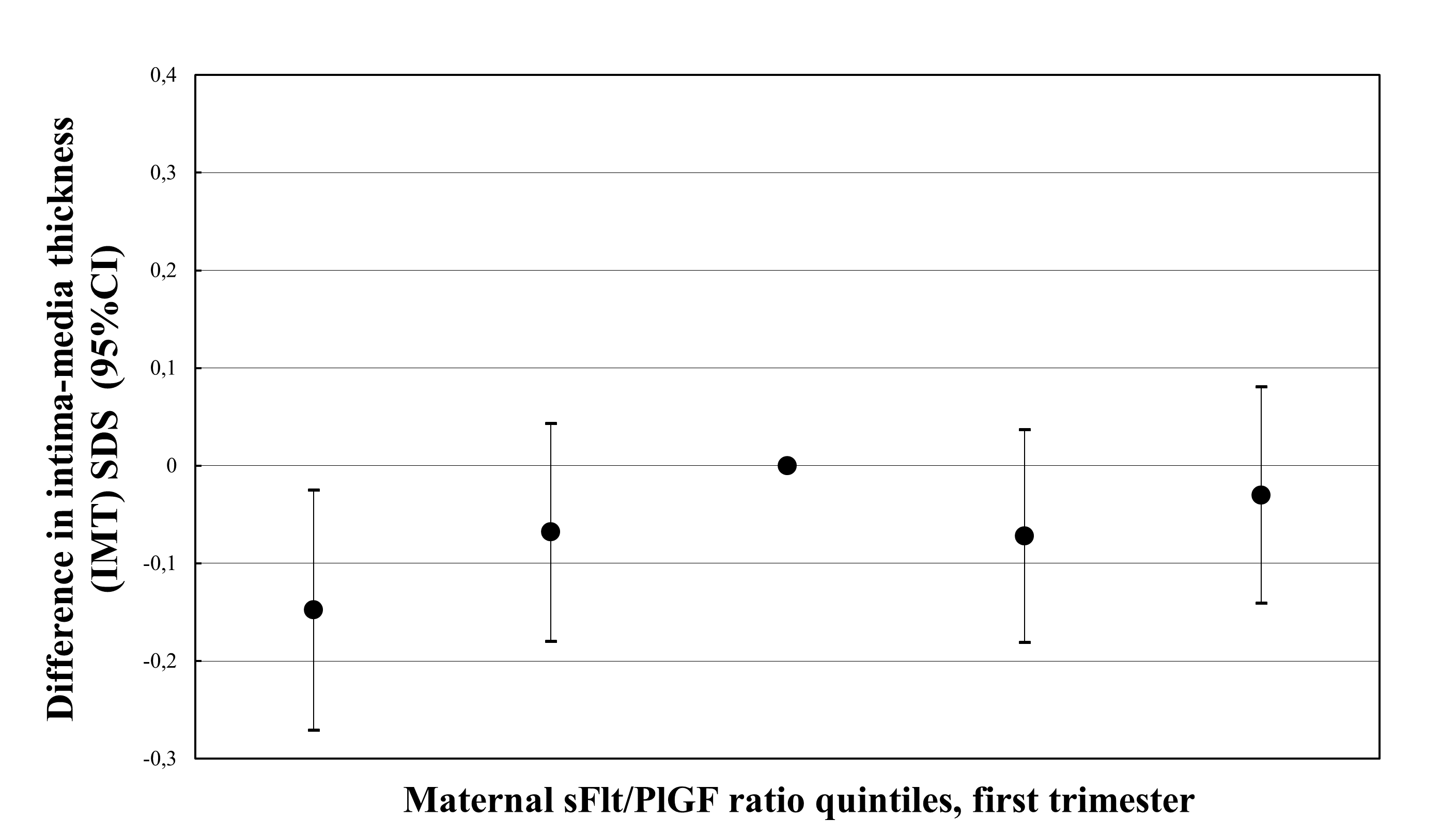

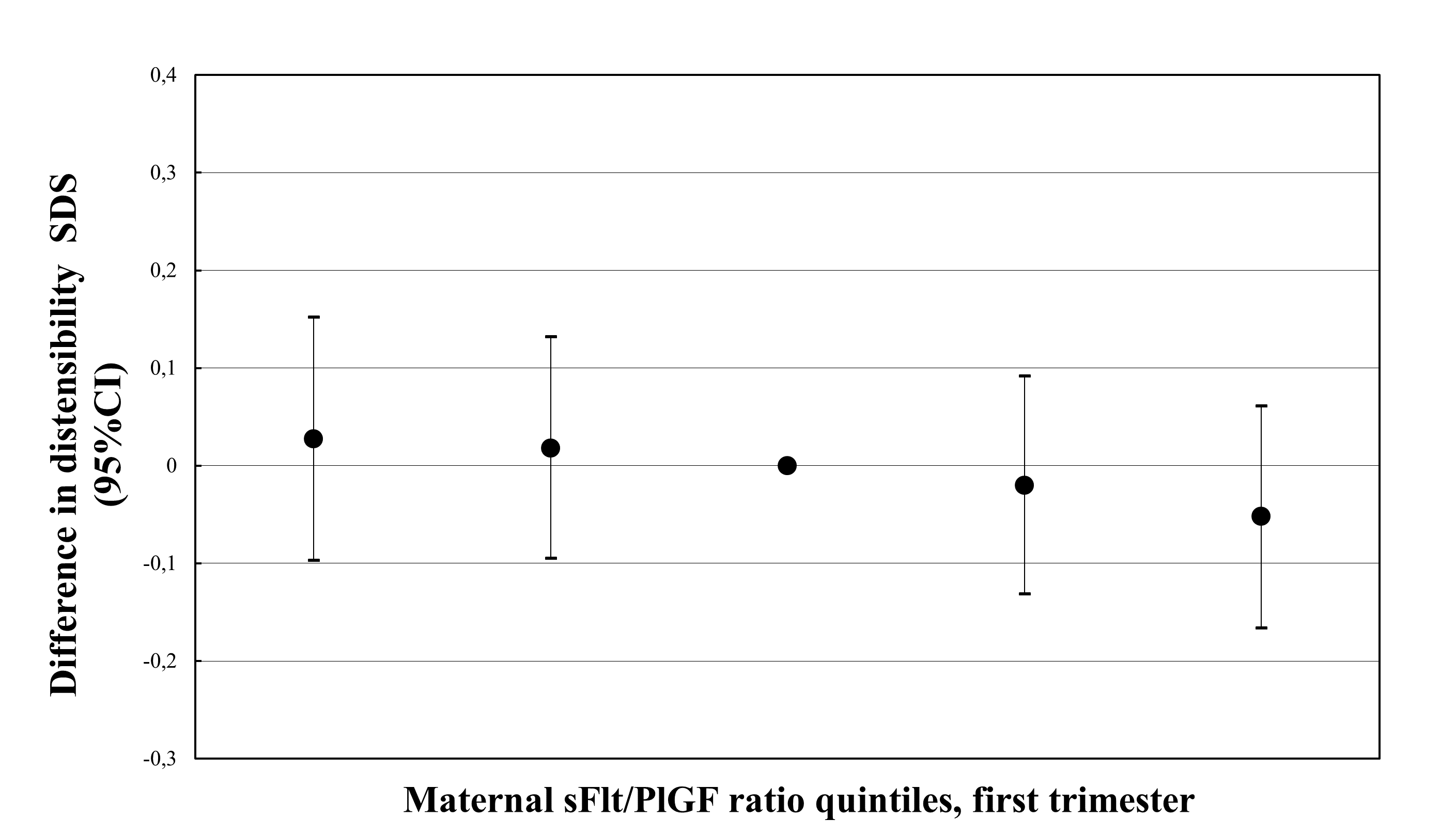


Regression analysis with childhood systolic blood pressure, diastolic blood pressure, carotid intima media thickness and carotid distensibility as dependent variables and maternal sFlt-1/PlGF ratio in first trimester as independent variable. Each point shows the strength of association (±95% CI) sFlt-1/PlGF ratio was divided in quintiles. The third quintile was the reference group. There was an adjustment for gestational age at intake, gestational age at blood sampling, educational level, ethnicity, parity, prepregnancy BMI, blood pressure, smoking, alcohol consumption, folic acid supplement use and child’s age and sex. 1): first trimester sFlt-1/PlGF ratio and childhood systolic blood pressure 2): first trimester sFlt-1/PlGF ratio and childhood diastolic blood pressure 3): first trimester sFlt-1/PlGF ratio and carotid intima media thickness 4): first trimester sFlt-1/PlGF ratio and carotid distensibility

**Figure S5** Regression analysis with maternal second trimester PlGF concentrations in quintiles


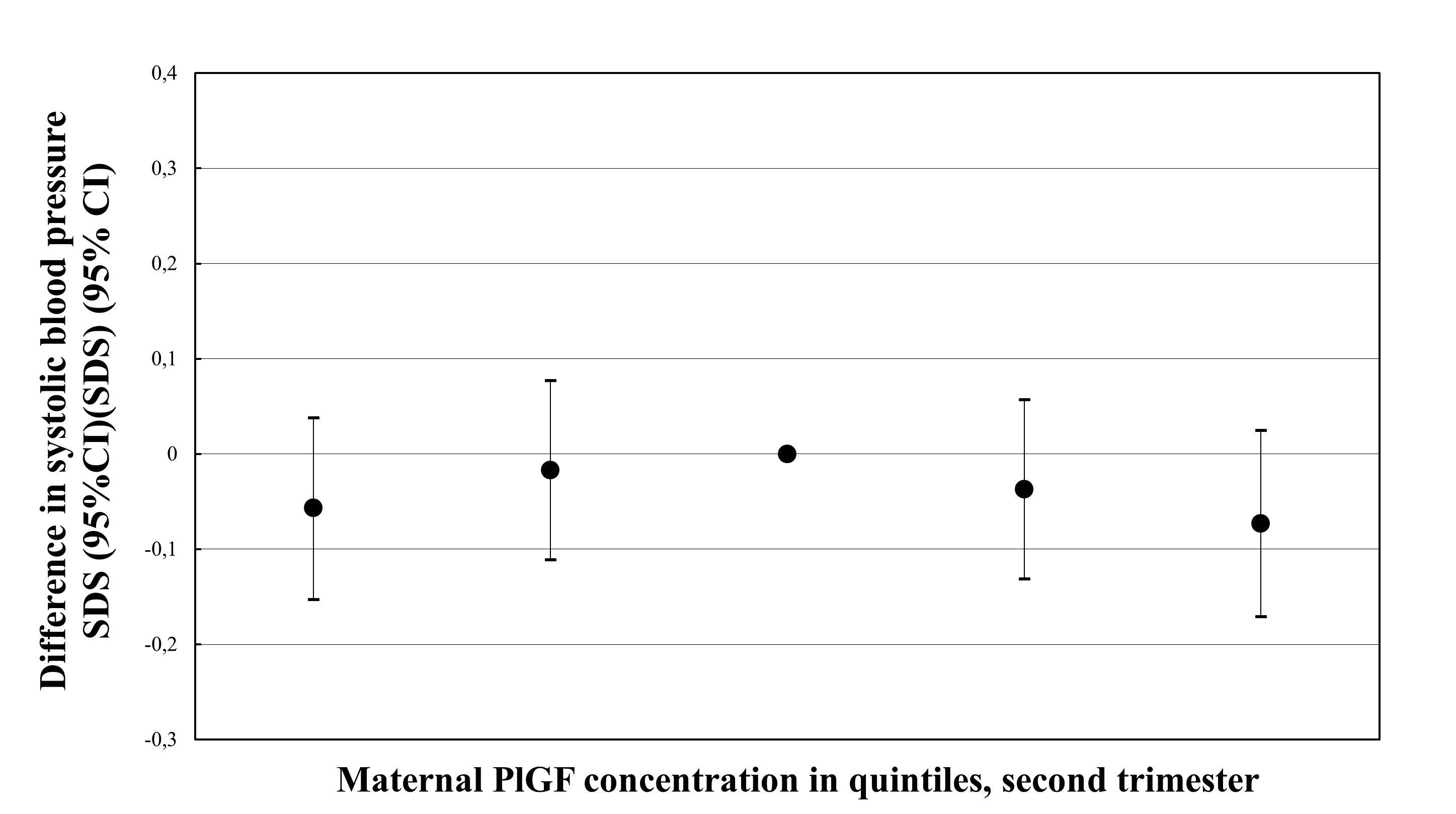

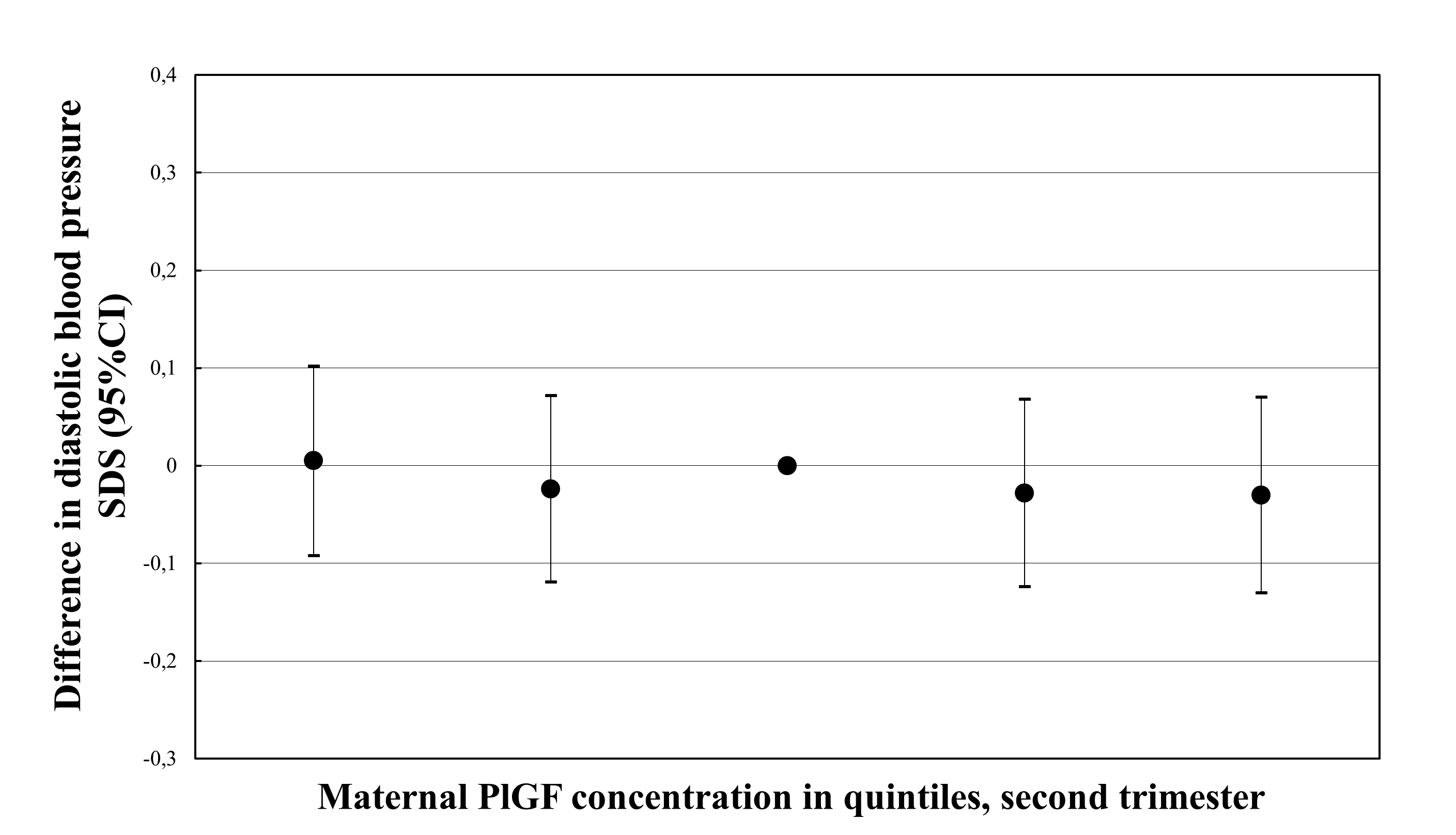


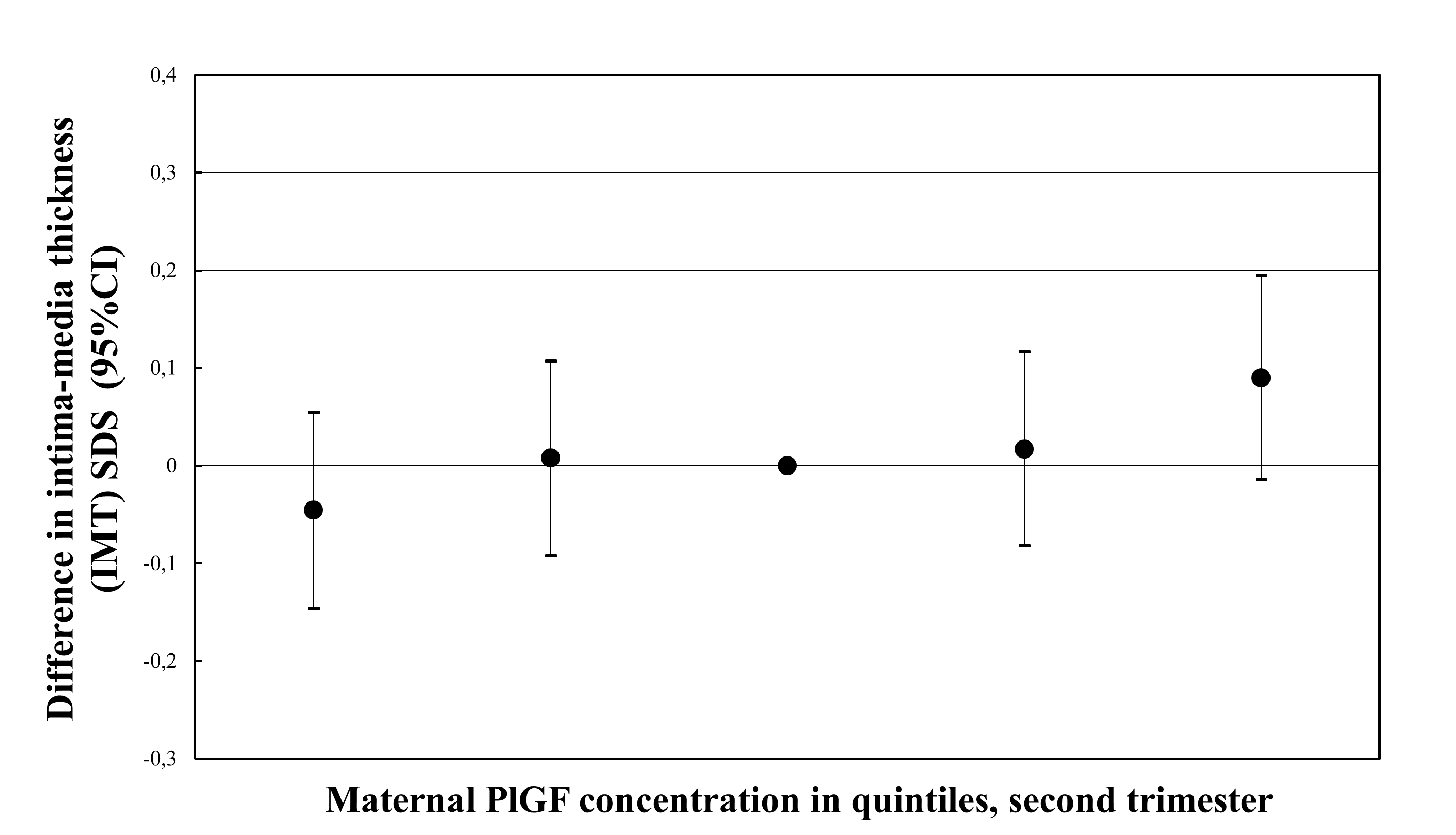

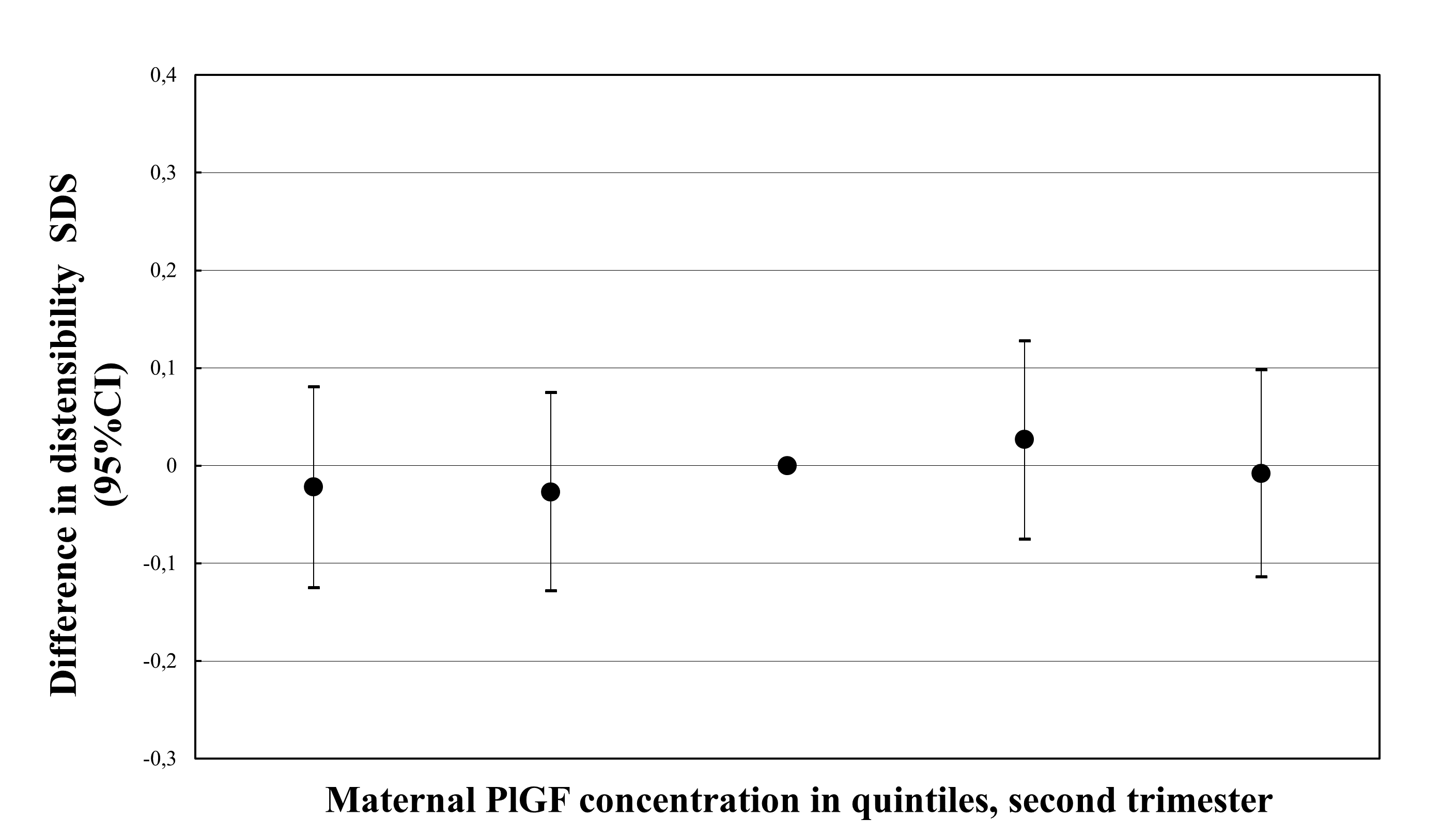


Regression analysis with childhood systolic blood pressure, diastolic blood pressure, carotid intima media thickness and carotid distensibility as dependent variables and maternal PlGF in second trimester as independent variable. Each point shows the strength of association (±95% CI) PlGF was divided in quintiles. The third quintile was the reference group. There was an adjustment for gestational age at intake, gestational age at blood sampling, educational level, ethnicity, parity, prepregnancy BMI, blood pressure, smoking, alcohol consumption, folic acid supplement use and child’s age and sex. 1): second trimester PlGF and childhood systolic blood pressure 2): second trimester PlGF and childhood diastolic blood pressure 3): second trimester PlGF and carotid intima media thickness 4): second trimester PlGF and carotid distensibility

**Figure S6** Regression analysis with maternal second trimester sFlt-1 concentrations in quintiles


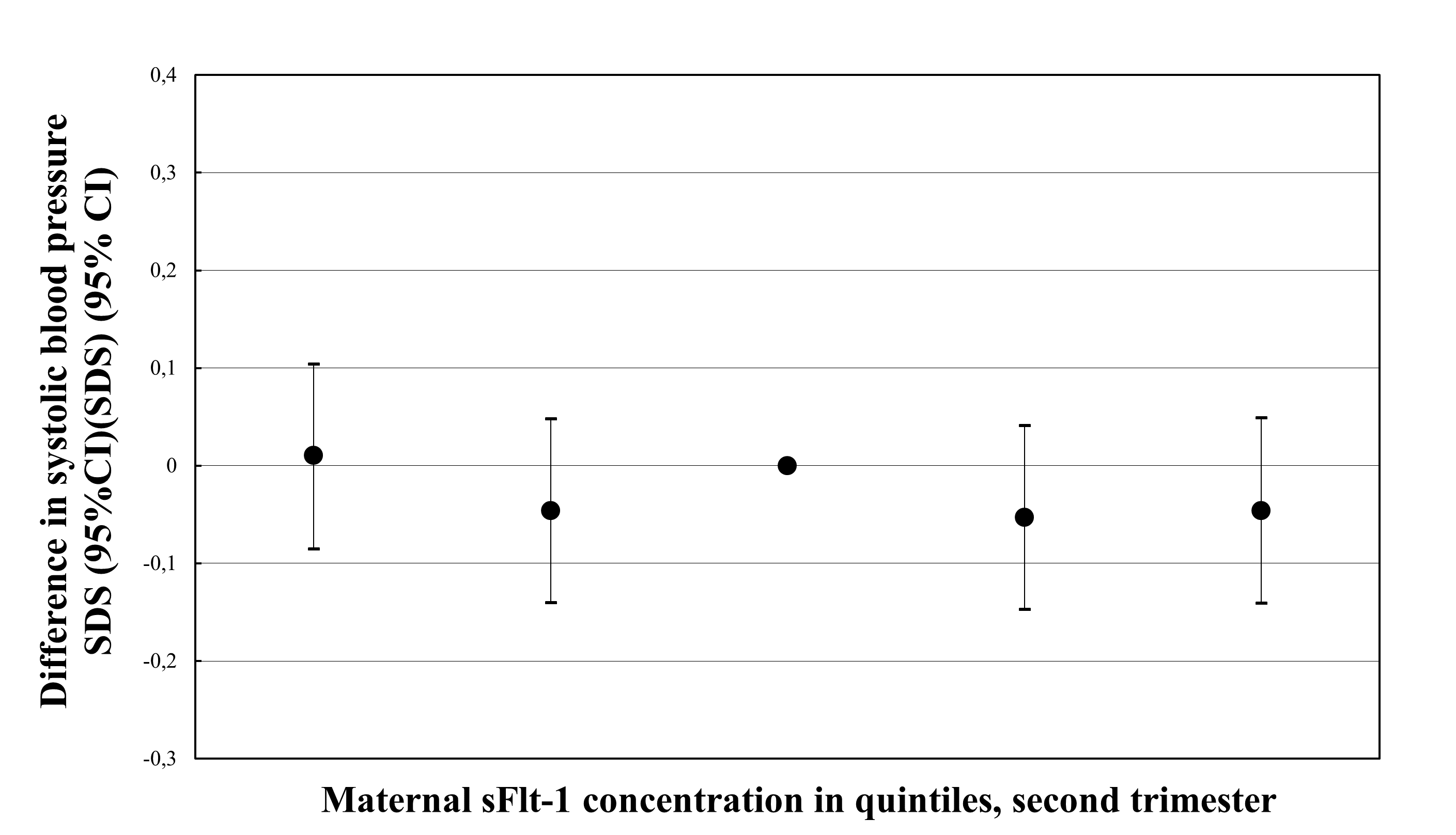

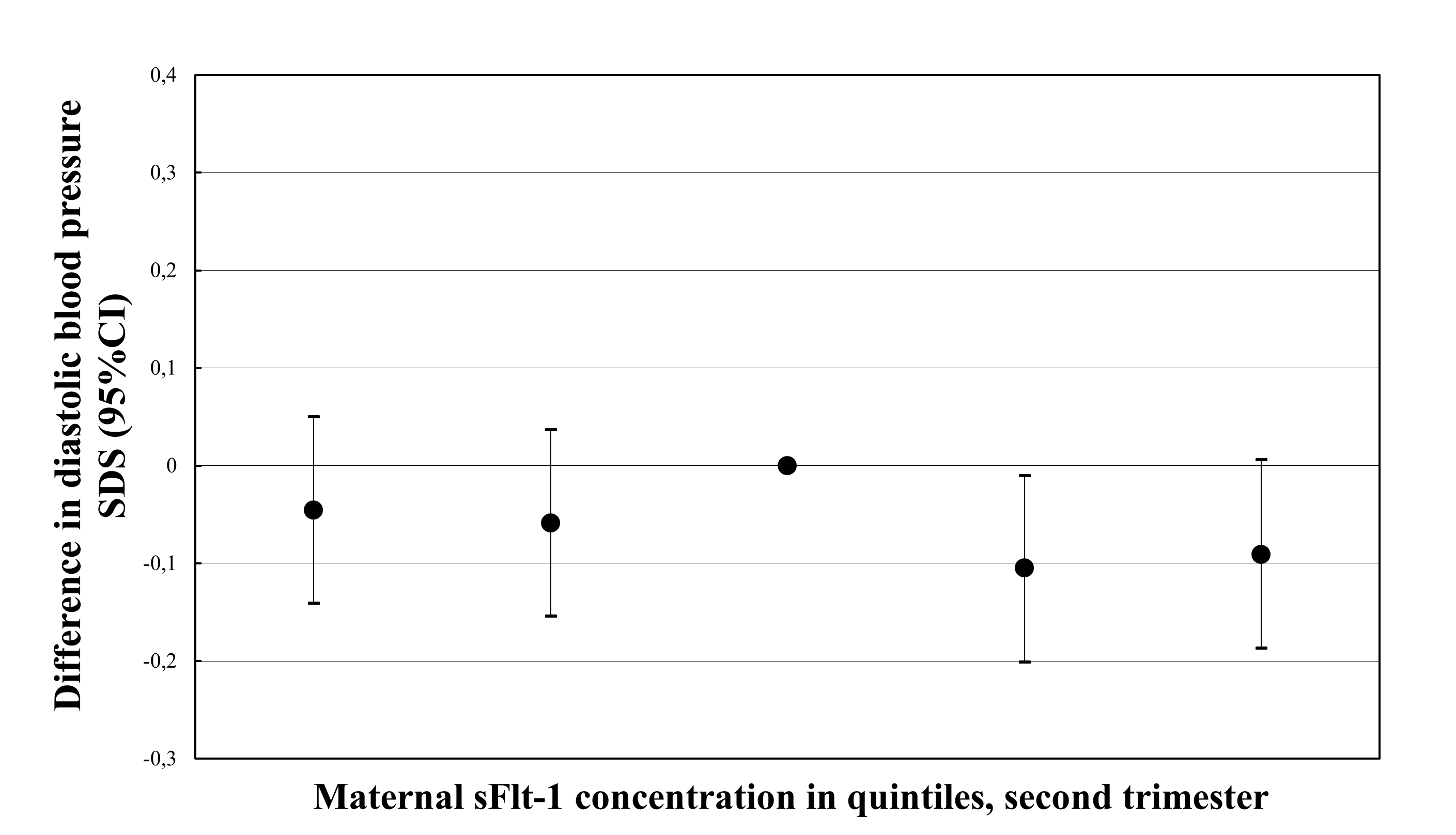


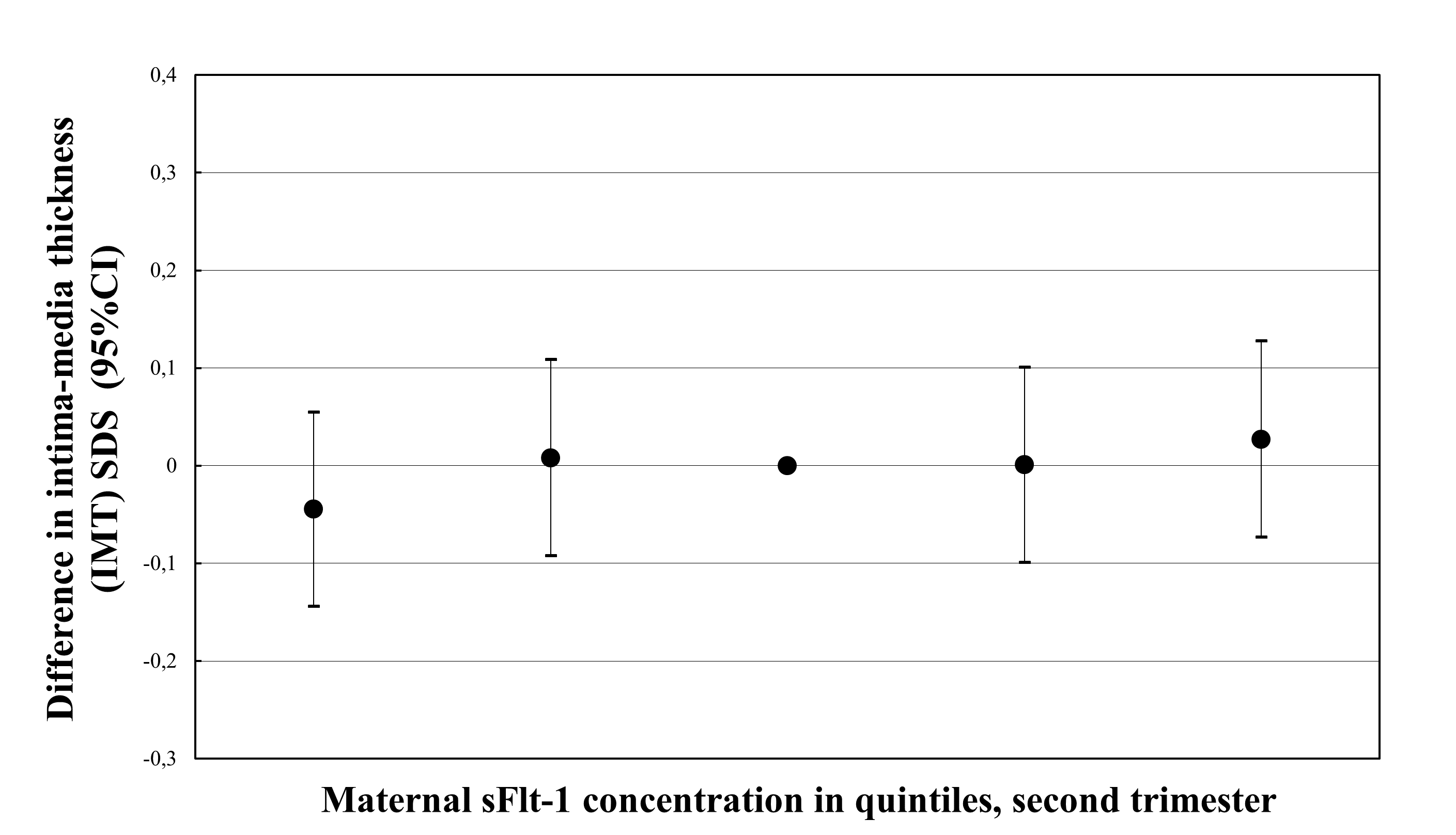

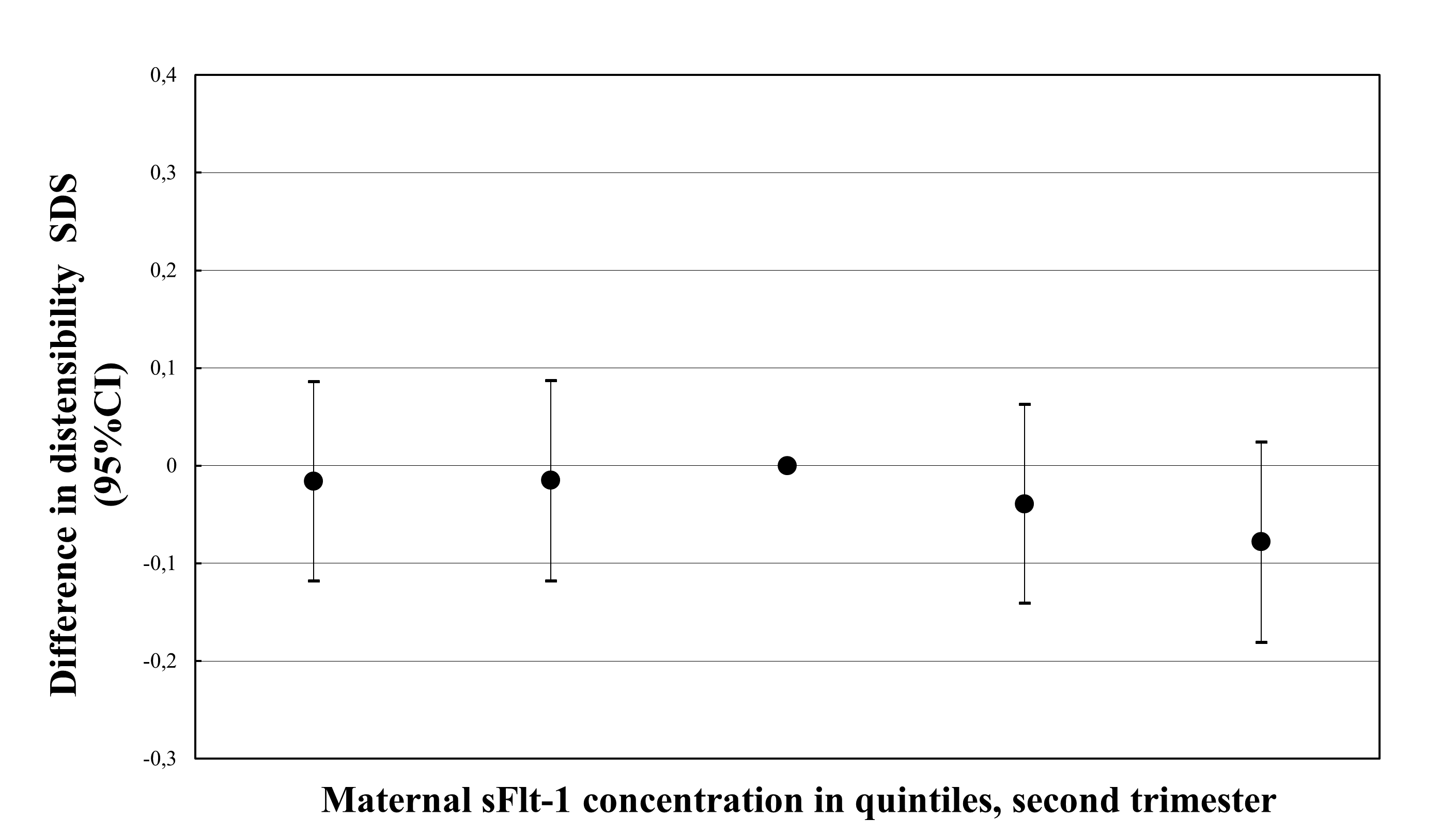


Regression analysis with childhood systolic blood pressure, diastolic blood pressure, carotid intima media thickness and carotid distensibility as dependent variables and maternal sFlt-1 in second trimester as independent variable. Each point shows the strength of association (±95% CI) sFlt-1 was divided in quintiles. The third quintile was the reference group. There was an adjustment for gestational age at intake, gestational age at blood sampling, educational level, ethnicity, parity, prepregnancy BMI, blood pressure, smoking, alcohol consumption, folic acid supplement use and child’s age and sex. 1): second trimester sFlt-1 and childhood systolic blood pressure 2): second trimester sFlt-1 and childhood diastolic blood pressure 3): second trimester sFlt-1 and carotid intima media thickness 4): second trimester sFlt-1 and carotid distensibility

**Figure S7** Regression analysis with maternal second trimester PlGF/sFlt-1 ratio in quintiles


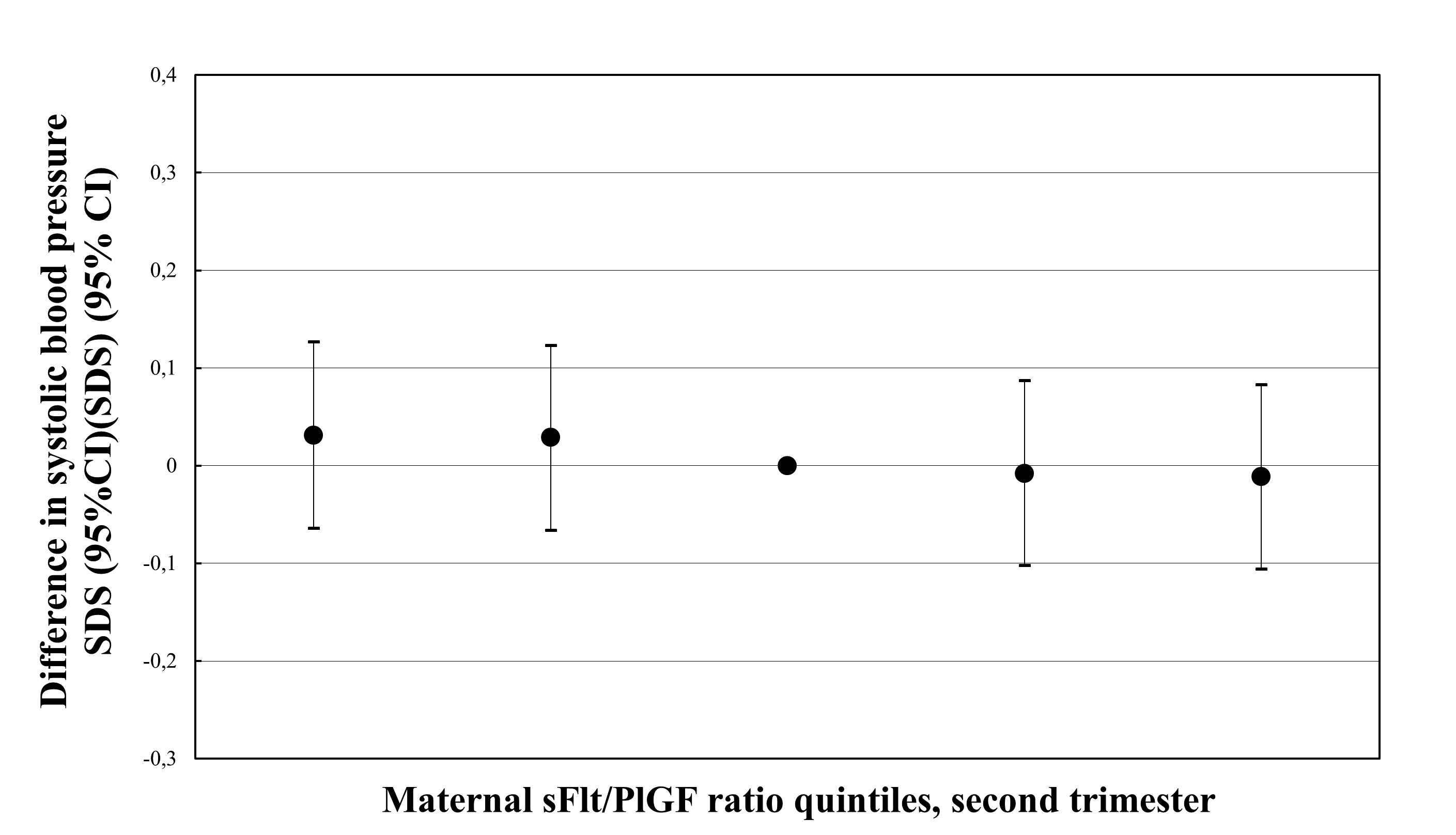

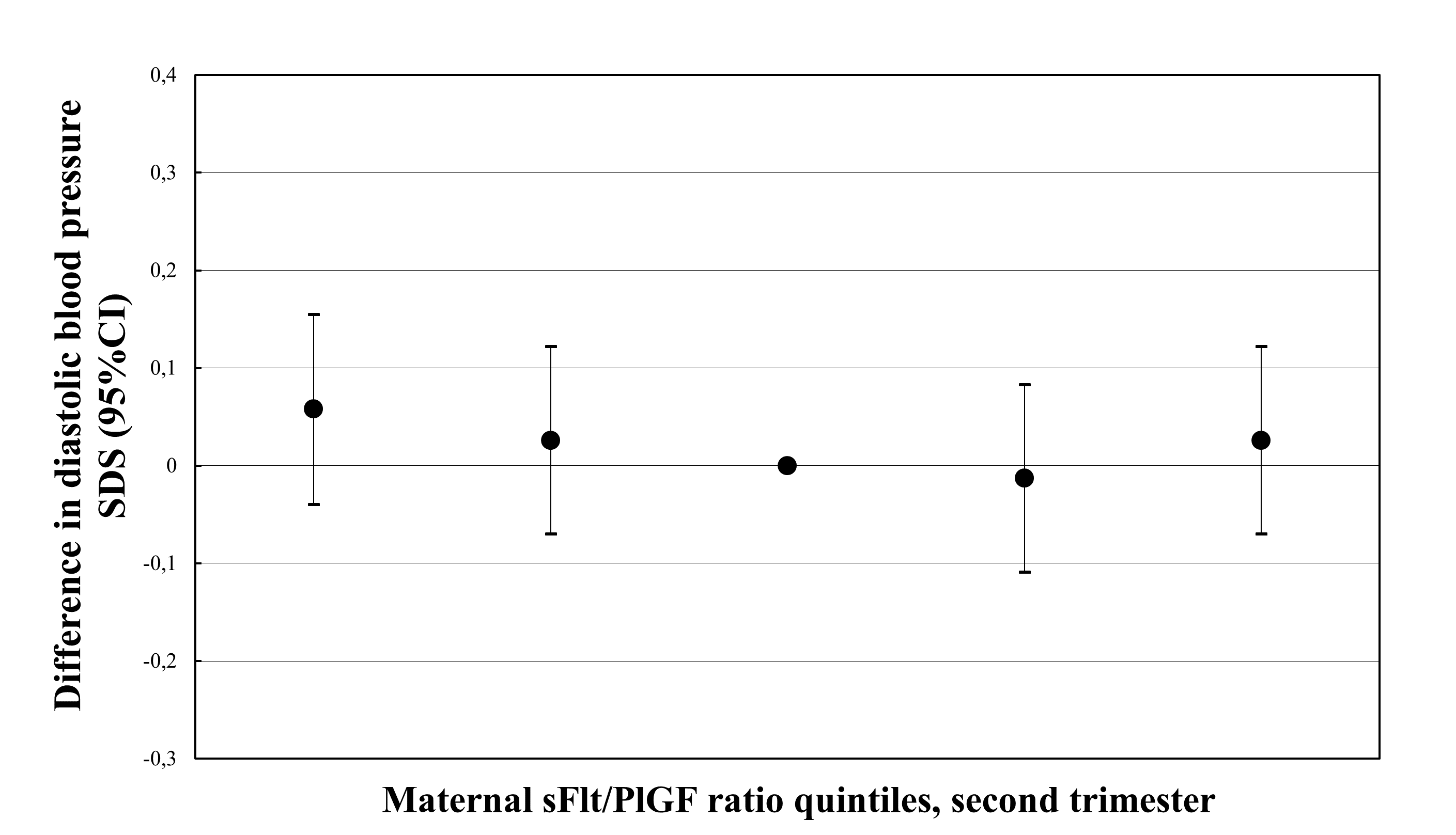


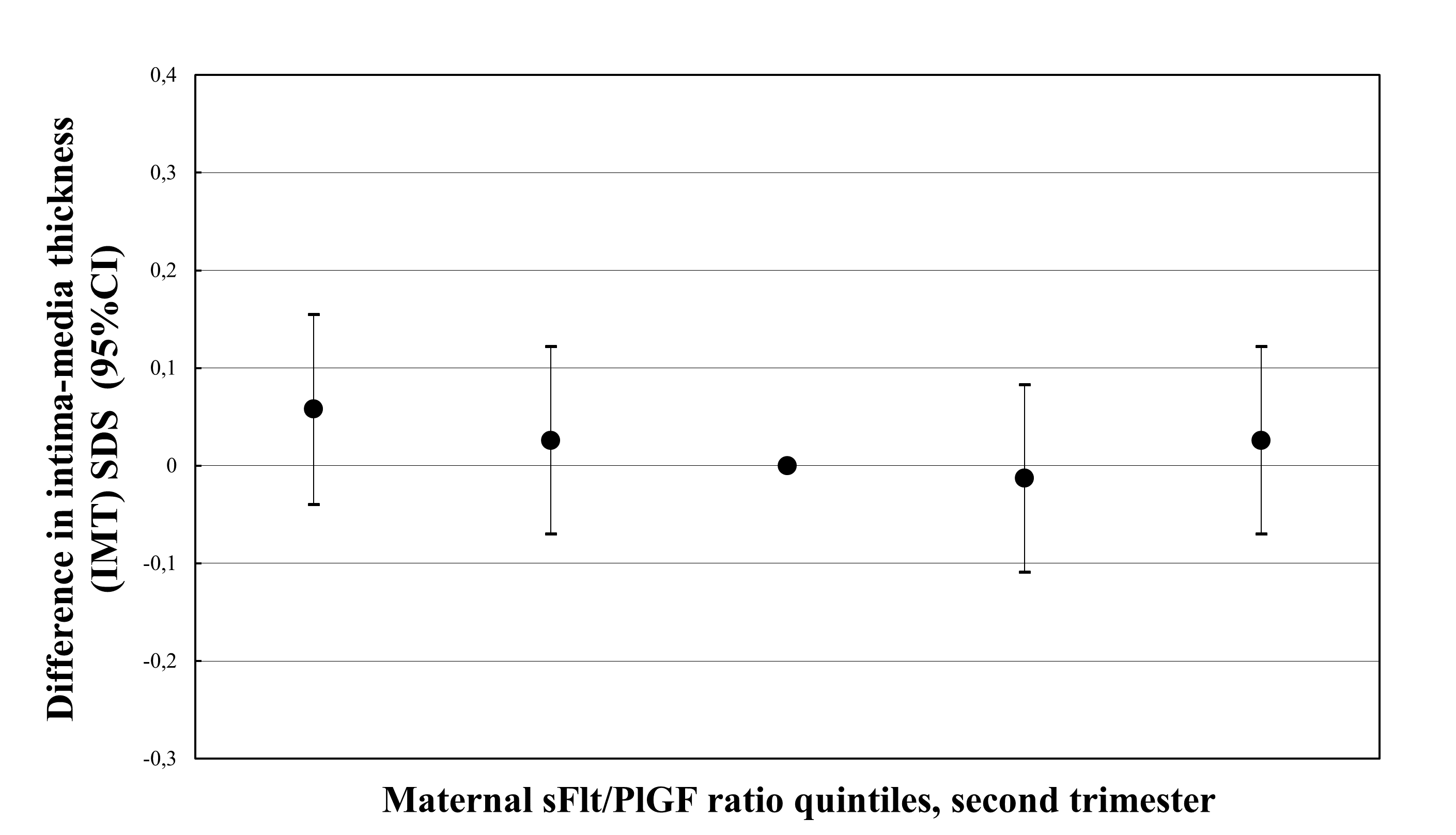

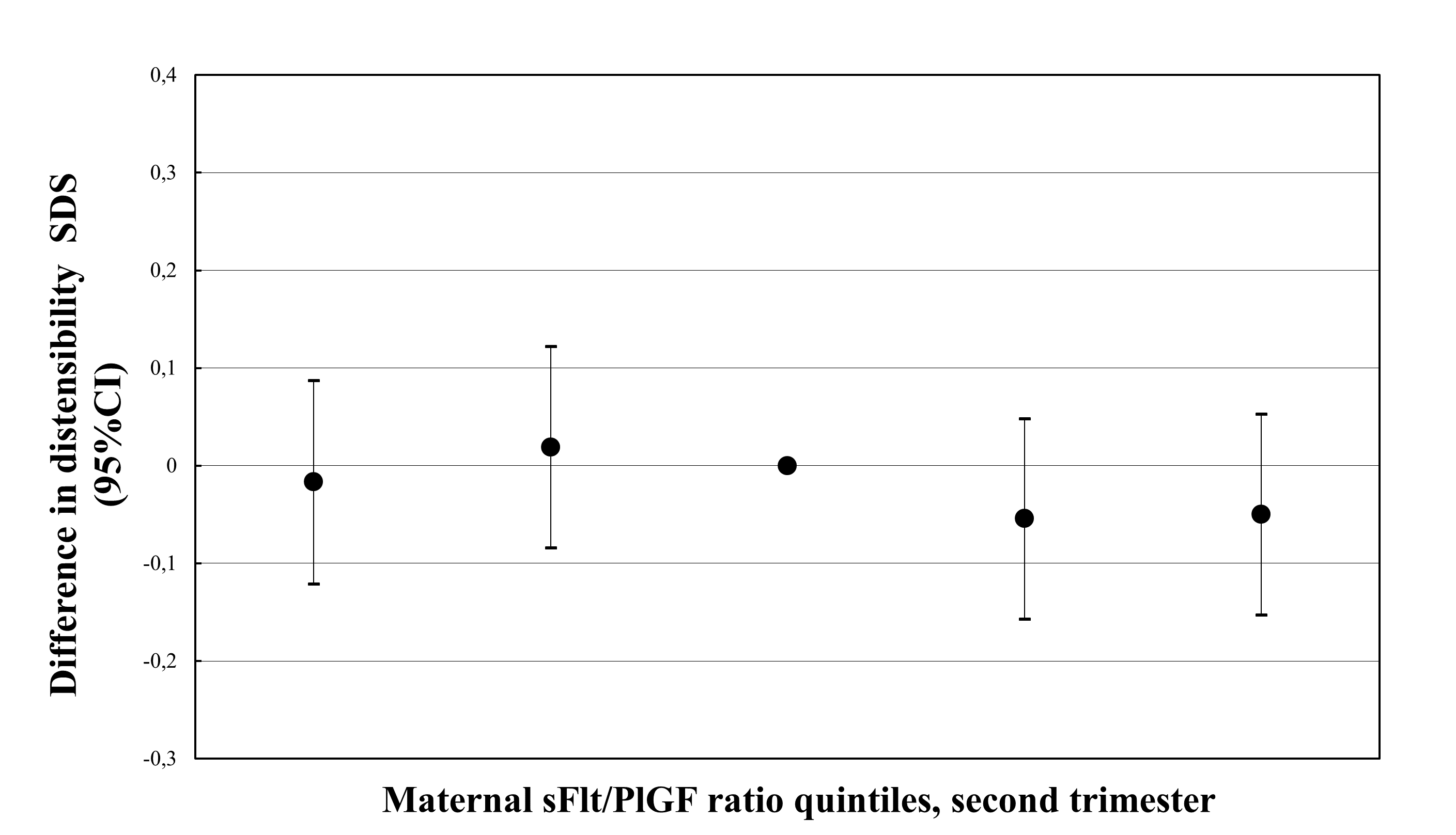


Regression analysis with childhood systolic blood pressure, diastolic blood pressure, carotid intima media thickness and carotid distensibility as dependent variables and maternal sFlt-1/PlGF ratio in second trimester as independent variable. Each point shows the strength of association (±95% CI) sFlt-1/PlGF ratio was divided in quintiles. The third quintile was the reference group. There was an adjustment for gestational age at intake, gestational age at blood sampling, educational level, ethnicity, parity, prepregnancy BMI, blood pressure, smoking, alcohol consumption, folic acid supplement use and child’s age and sex. 1): second trimester sFlt-1/PlGF ratio and childhood systolic blood pressure 2): second trimester sFlt-1/PlGF ratio and childhood diastolic blood pressure 3): second trimester sFlt-1/PlGF ratio and carotid intima media thickness 4): second trimester sFlt-1/PlGF ratio and carotid distensibility.
